# A novel targeted multifunctional nanoplatform for visual chemo-hyperthermia synergy therapy on metastatic lymph nodes via lymphatic delivery

**DOI:** 10.1186/s12951-021-01186-8

**Published:** 2021-12-20

**Authors:** Weiwei Liu, Xiaoping Ye, Lingyun He, Juan Cheng, Wenpei Luo, Min Zheng, Yaqin Hu, Wei Zhang, Yang Cao, Haitao Ran, Lu Yang

**Affiliations:** 1 Chongqing Key Laboratory of Ultrasound Molecular Imaging, Institute of Ultrasound Imaging, Department of Ultrasound, The Second Affiliated Hospital of Chongqing Medical University, Chongqing, 400010 People’s Republic of China; 2 Department of Breast and Thyroid, The Second Affiliated Hospital of Chongqing Medical University, Chongqing, 400010 People’s Republic of China

**Keywords:** Carbon nanoparticles, Metastatic lymph nodes, Photonic hyperthermia, Chemotherapy, Nanomedicine, Targeted therapy, Molecular imaging

## Abstract

**Background:**

Distant metastasis to vital organs is the major contributor to breast cancer mortality, and regional lymph node metastasis is an important facilitator of distant metastasis and recurrence in this cancer. The early diagnosis and precise treatment of lymph node metastasis are crucial for staging and prognosis in breast cancer. Herein, we report a visualized precision medicine nanoplatform of metastatic lymph nodes for ultrasonic/photoacoustic (US/PA) dual modal imaging-guided in situ targeted hyperthermia-combined chemotherapy.

**Results:**

Carbon nanoparticles (CNs), approved by the China Food and Drug Administration, were loaded with docetaxel and rationally combined with anti-hypoxia-inducible factor 1α antibody-modified poly (lactic-co-glycolic acid) (PLGA) nanoparticles to achieve the combination of passive targeting at the lymph nodes and intracellular targeting at HIF 1α factor. The accumulation and retention of nanoparticles in metastatic lymph nodes via lymphatic delivery were enhanced. Docetaxel could be effectively offloaded by CNs that have active carbon nanoparticles, and the PLGA membrane prevented drug leakage. The nanoparticles exhibited excellent photothermal performance with a photothermal conversion efficiency of 28.9%, killing tumor cells in metastatic lymph nodes through hyperthermia. In vitro and in vivo systematic evaluations revealed that hyperpyrexia triggered the rupture of nanoparticles caused by the phase transition of perfluorohexane, resulting in docetaxel release for achieving in situ hyperthermia-combined chemotherapy.

**Conclusions:**

The laser-triggered highly efficient in situ chemotherapy nanosystem achieves targeted synergistic chemo-hyperthermia treatment of metastatic lymph nodes, and lymphatic delivery represents a strategy to avoid additional injury caused by drugs entering the blood circulation.

**Graphical Abstract:**

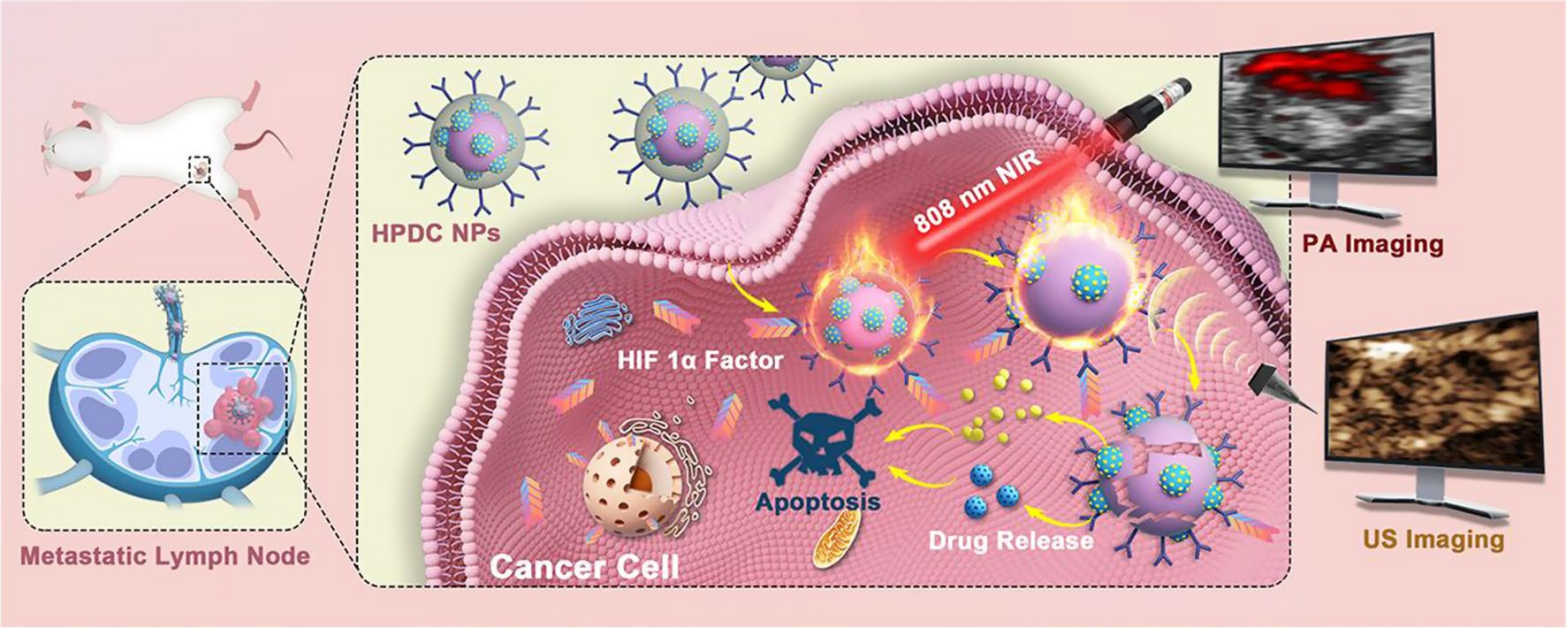

**Supplementary Information:**

The online version contains supplementary material available at 10.1186/s12951-021-01186-8.

## Introduction

Breast cancer remains one of the gravest and lethal diseases that seriously threaten women’s health nowadays, whose incidence has surpassed that of lung cancer and ranked first among all tumors in 2020 [1–3]. Distant metastasis in breast cancer mainly occurs in vital organs including liver, lung and brain through the regional lymph node metastasis, which is the primary cause of mortality [4–7]. The early diagnosis and precise treatment of lymph nodes have potential clinical value for staging and treatment prognosis in breast cancer [8–10]. Most traditional treatments for metastatic lymph nodes include surgery, chemotherapy, and radiation therapy [11–13], and surgical lymph node dissection is typically used for staging and removing metastatic lymph nodes [14, 15]. However, surgical dissection may severely damage the axillary lymphatic system, resulting in edema and motor dysfunction in the ipsilateral upper limb [16, 17]. Moreover, many conventional intravenously injected chemotherapeutic agents failed to show maximal efficacy because of their limited access to the lymphatic system [18]. With intravenous chemotherapy, the pathological complete remission rate of metastatic lymph nodes is very low [19] and causes serious systemic adverse effects [20], such as bone marrow suppression [21] and liver injury [22]. According to current guidelines, radiation therapy is necessary for patients with more than four metastatic lymph nodes [23], but this could aggravate surgery-related complications and other adverse effects of radiation such as pneumonia and dermatitis [24]. Lymph nodes are the sites where both the blood circulatory and lymphatic systems are indirectly connected [25, 26]. Therefore, in this study, we proposed to inject drugs subcutaneously guided by sentinel lymph node imaging, where drugs can passively target the lymph nodes at all levels via the lymphatic system and barely enter the blood circulation to efficiently reduce or even avoid additional injury. Using multimodal imaging for the precise visualization of metastatic lymph nodes [27–29], we aimed to achieve multimodal imaging guided synergistic chemo-hyperthermia therapy and evaluation of therapeutic effect [30, 31]. In addition, lymphatic injection-mediated passive targeting combined with antibody-mediated intracellular targeting can achieve precision therapy at the cellular level [32, 33]. We expect that this new therapeutic modality and route of administration will effectively avoid the adverse effects of surgery, chemotherapy, and radiotherapy.

An injectable suspension of carbon nanoparticles (CNs), approved by the China Food and Drug Administration as a lymphatic tracer [34, 35], is widely used for sentinel lymph node tracing in breast cancer with lymphatic tropism due to its efficient dyeing ability and favorable biosafety [36]. In addition, CNs have stable physical and chemical properties, and their porous honeycomb structure allows them to effectively adsorb various chemotherapeutic drugs and function as an excellent carrier [37]. It is worth noting that CNs have excellent near-infrared (NIR) absorption property, which indicates the potential application prospects of CNs in photoacoustic (PA) imaging-guided photothermal therapy [38–40]. Due to the great biocompatibility, superior drug loading potency, and excellent photothermal conversion efficiency, CNs injectable suspension is one of the most crucial materials with great potential for clinical translation to achieve chemo-hyperthermia synergy.

Oxygen plays a key substrate in cell metabolism, signal transduction and maintaining normal physiological functions, while hypoxia is the basic feature of the tumor microenvironment (TME) [41–43]. Hypoxia-inducible factor 1 (HIF 1) is an important transcription factor that regulates oxygen homeostasis [44, 45] by inducing the expression of genes that mediate the adaptive response of cells to hypoxia [46, 47]. Under normoxic conditions, HIF 1α undergoes hydroxylation on particular prolyl residues making the synthesized HIF 1 protein highly susceptible to the intracellular oxygen-dependent ubiquitin protease degradation pathway [48]. However, under hypoxic conditions, the HIF 1α regulatory subunit is stable [49]. Thus, the presence of HIF 1α acts as a marker of the hypoxic tumor microenvironment [50].

**Scheme 1:**
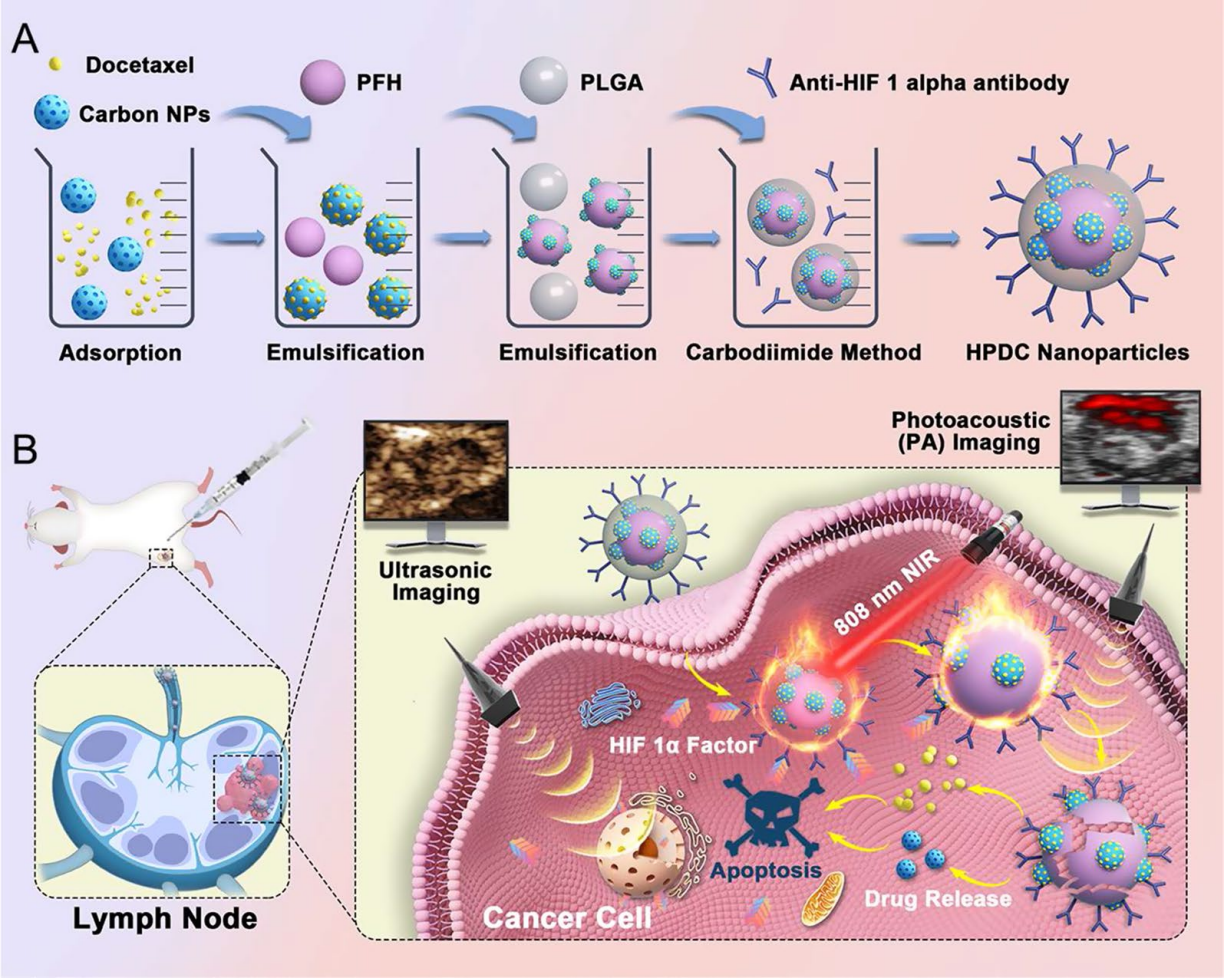
**A** Schematic diagram of the manufacturing process of HPDC NPs. **B** The corresponding synergistic effect of 808 nm laser-triggered hyperthermia and DOC-mediated chemotherapy

We rationally loaded CNs with docetaxel (DOC) and perfluorohexane (PFH) and combined them with anti-HIF 1α antibody-modified PLGA nanoparticles (denoted as HPDC NPs) to achieve US/PA dual imaging-guided and laser-triggered in situ DOC release for both passive and intracellular targeted photothermal therapy combined with DOC-induced chemotherapy (Scheme 1). HPDC NPs accumulated at metastatic lymph node sites via passive targeting by lymphatic injection and intracellular targeting at HIF 1α factor mediated by anti-HIF 1α antibody. After endocytosis, HPDC NPs with excellent photothermal conversion efficiency represented obvious temperature enhancement upon exposure to near infrared-I (NIR − I) laser for US/PA dual modal imaging-monitored photothermal therapy. Hyperpyrexia triggered the rupture of HPDC NPs caused by the phase transition of PFH, resulting in DOC release for achieving the in situ chemotherapeutic effects. This study provides a visualized precision medicine nanoplatform of metastatic lymph nodes and a new administration method for realizing previously envisaged US/PA imaging-guided and laser-triggered in situ hyperthermia-combined chemotherapy, which could be a new alternative strategy for metastatic lymph nodes. More importantly, the materials used to prepare the NPs have been approved for biomedical applications due to their biosafety, facilitating their clinical translation.

## Experimental

### Materials

CNs (50 mg mL^−1^) and DOC were purchased from Lummy (China). Anti-HIF 1α antibody was purchased from Abcam (UK, MW = 93,000). PLGA (50:50, MW = 12,000), PFH, 1-(3-dimethylaminopropyl)-3-ethylcarbodiimide hydrochloride (EDC), 2-(N-morpholino) ethanesulfonic acid hydrate (MES), N-hydroxy succinimide (NHS), polyvinyl alcohol (PVA, MW = 25,000), 1,1- dioctadecyl-3,3,3′,3′-tetramethylindocarbocyanine perchlorate (DiI), and fluorescein isothiocyanate (FITC) were purchased from Sigma-Aldrich Co., Ltd. (USA). Calcein acetoxymethyl ester (Calcein-AM), propidium iodide (PI), and 4′, 6-diamidino-2-phenylindole (DAPI) were ordered from JenKem Technology Co., Ltd. (Beijing). Annexin V-FITC apoptosis detection kit and cell counting kit-8 (CCK-8) were purchased from Dojindo Co., Ltd (Japan). Fetal bovine serum, Roswell Park Memorial Institute 1640 (RPMI 1640), phosphate buffer saline (PBS), and 0.25% trypsinization-EDTA solution were purchased from Thermo Fisher Scientific (USA). Dichloromethane (CH_2_Cl_2_) and dimethyl sulfoxide (DMSO) were purchased from Chuandong Chemical Co. Ltd (Chongqing, China). All other reagents used were of analytical grade without further purification. The Millipore water purification system was used to obtain deionized (DI) water.

### Characterization

Transmission electron microscopy (TEM) images were acquired using a JEM-2100F transmission electron microscope. The zeta potential and dynamic light scattering (DLS) measurements were made using the Zeta sizer series (Nano ZS90, Malvern Instrument Ltd.). UV–vis-NIR absorption spectra were obtained on a UV-3101PC Shimadzu spectrometer. High-performance liquid chromatography (HPLC) analysis was performed on an LC-2010A HT (Shimadzu, Kyoto, Japan). Confocal laser scanning microscopy (CLSM) images were obtained using FV1000 (Olympus Company, Japan). Cell apoptosis and cellular endocytosis of NPs were examined by flow cytometry analysis (BD LSRFortessa, USA). Photothermal hyperthermia performance was evaluated using 808 nm multimode pump laser irradiation (Shanghai Connect Fiber Optics Co. Ltd.). Photoacoustic (PA) images were obtained using the Vevo LAZR PA system (VisualSonics Company, Canada).

### Synthesis of PFH-DOC-CNs nanoparticles

PFH-DOC-CNs nanoparticles (denoted as PDC NPs) were fabricated following a revised three-step emulsion process. Briefly, 200 μL of CNs (10 mg) suspension and 2 mg of DOC were mixed under the ultrasonic probe with a power of 130 W for 1 min and transferred to an NHWY-200F horizontal thermostat oscillator (China) and shaken for 24 h at 37 °C with a speed of 120 rpm. Next, 200 μL of PFH was added and mixed with 2 mL dichloromethane, containing 50 mg dissolved PLGA. Subsequently, the mixture suspension was emulsified using an ultrasonic probe (130 W for 4 min) in an ice bath. For the third emulsification, 10 mL of PVA solution (w/v = 5%) was homogenized using FJ300-SH (China) for 3 min in an ice bath. Then, 12 mL of isopropyl alcohol solution (w/v = 2%) was added into the emulsion above, which was stirred with a magnetic stirrer (HJ-1, Ronghua, China) in an ice bath for 2 h. After dichloromethane volatilized, the emulsion was centrifuged at 11,000 rpm (5 min, 4 °C) and washed three times with DI water to obtain PDC NPs. The obtained PDC NPs were fully dispersed in DI water and saved at 4 °C until further use.

### Synthesis of HPDC NPs

HPDC NPs were synthesized following the carbodiimide technique. Briefly, 200 μL of PDC NPs (1 mg mL^−1^) was added into 10 mL of MES solution (0.1 mol L^−1^, pH 5.0) and the excess EDC and NHS (molar ratio = 2:1) were introduced in sequence. The mixed solution was placed on a horizontal thermostat oscillator for 2 h (0 °C, 120 rpm) and then centrifuged at 11,000 rpm (4 °C for 5 min) and washed with DI water three times. After that, the activated PDC NPs were suspended in MES solution (10 mL, 0.1 mol L^−1^, pH 8.0), in which the excess anti-HIF 1α antibody (100 μL, 1 mg mL^−1^, MW = 93,000) was added. Subsequently, the mixture above was shaken for another 2 h. The resulting HPDC NPs were centrifuged (11,000 rpm for 5 min) and washed twice. These obtained HPDC NPs were dispersed in DI water and stored at 4 °C. The DiI-labeled NPs and NPs without CNs, DOC or PFH were fabricated by the same methods.

### Drug loading and release performance

First, 1 mL of HPDC NPs (5 mg mL^−1^) were centrifuged and dissolved in 5 mL of DMSO for demulsification. The absorption curve of CNs was conducted through an ultraviolet spectrophotometer. The DOC concentration was obtained using HPLC. The drug encapsulation efficiency of drugs (DEE) and drug loading capacity (DLC) were calculated by the following formulas: DEE (%) = (Wm/Wt) % and DLC = (Wm/Ws) % (Wm: weight of CNs or DOC in HPDC NPs, Wt: total weight of CNs or DOC planned input in HPDC NPs, and Ws: weight of HPDC NPs).

The NIR laser-activated drug release behavior was demonstrated in three different groups: HPDC NPs group, HPDC NPs + Laser group, and HDC NPs (HPDC NPs without PFH) + Laser group. In a nutshell, 5 mg of HPDC NPs and 5 mg of HDC NPs were dispersed in PBS (1 mL) and placed in three independent dialysis bags (4–8 kDa). After that, these three bags were immersed in corresponding flasks containing 100 mL of simulated body fluid at 37 °C (rotation speed 120 rpm). These bags containing HPDC NPs and HDC NPs were shaken 2 h before laser irradiation (1.0 W cm^−2^ for 5 min) and then shaken for another 2 days. Next, 1 mL of aliquots were taken from the three groups at different times (0, 1, 2, 3, 4, 8, 12, 24, and 48 h), and 1 mL of simulated body fluid was replaced into the flasks. The amount of DOC released from HPDC NPs and HDC NPs was directly quantified by HPLC analysis.

### In vitro photothermal performance of HPDC NPs

HPDC NPs solution of various concentrations (25, 50, 100, 200, 400, and 800 ppm) were exposed to laser irradiation (1.0 W cm^−2^). HPDC NPs aqueous solution was irradiated by NIR − I laser irradiation at variable power intensities (0.25, 0.5, 0.75, 1.0, 1.25, and 1.5 W cm^−2^), and variations in the temperature of HPDC NPs exposed to NIR − I laser irradiation were monitored by infrared (IR) thermal imaging.

### Cell culture

Cellular and animal experiments were performed using Walker256 breast cancer cells. The tumor cells were obtained from the Shanghai Institute of Cells, Chinese Academy of Sciences. In a humid atmosphere containing 5% carbon dioxide at 37 °C, Walker256 cells were cultivated in RPMI 1640 with 10% fetal bovine serum and 1% streptomycin/penicillin.

### Cellular cytotoxicity evaluation

Walker256 cells were seeded in a 96-well plate (1 × 10^4^ per well) and co-incubated with HPDC NPs at varying concentrations for different duration of 12, 24, or 48 h, respectively. The cell viability was determined using the standard CCK-8 assay.

### Synergistic chemo-hyperthermia therapy in vitro

Walker256 cells were planted into 96-well plates (1 × 10^4^ per well). Then, six groups of cancer cells were established: control, laser only, DOC only, HPDC NPs only, HPC NPs + NIR − I laser, and HPDC NPs + NIR − I laser (1.0 W cm^−2^ for 5 min). The efficiency of synergistic chemo-hyperthermia was verified by the CCK-8 viability assay.

### Live-dead cell staining assay

Walker256 tumor cells were planted in 35 mm confocal dishes and subjected to different treatments: control, laser only, DOC only, HPDC NPs only, HPC NPs + NIR − I laser, and HPDC NPs + NIR − I laser (1.0 W cm^−2^) groups. The power density was 1.0 W cm^−2^ and the irradiation time was 5 min. Then, these dishes were washed three times via PBS and added 15 μL of Calcein-AM and 10 μL of PI for staining cells (20 min). Lastly, the dishes were washed three times by PBS and then observed on CLSM system.

### Apoptosis assays

Walker256 cells were plated in 6-well plates (4 × 10^5^ per well), and the adherent tumor cells were subjected to different treatments: control, 808 nm laser only, DOC only, HPDC NPs only, HPC NPs + 808 nm laser, and HPDC NPs + 808 nm laser (1.0 W cm^−2^) groups. Then, the cells were digested by trypsinization and centrifuged at 1000 rpm for 5 min. The precipitates obtained were resuspended in PBS and analyzed by flow cytometry after labeling with Annexin V-FITC and PI (20 min).

### Intracellular endocytosis of HPDC NPs

Walker256 tumor cells were seeded into 35 mm confocal dishes and incubated with DiI-labeled HPDC NPs for different durations (0.25, 0.5, 1, and 2 h). Then, the cell nuclei were stained by DAPI (500 μL, 10 min). Walker256 cells were washed three times with PBS and then observed by CLSM.

Walker256 cancer cells were planted into 6-well plates (4 × 10^5^ per well) for 24 h. The cell culture medium was replaced by DiI-labeled HPDC NPs, and then HPDC NPs were incubated with tumor cells for different durations (0.25, 0.5, 1, and 2 h). Then, Walker256 cells were digested by trypsinization, resuspended in PBS, and analyzed by flow cytometry.

### CircRNAs profiling analysis

Before image recognition and base recognition, premier reads were harvested from Illumina Novaseq 6000 sequencer. Cutadapt software was used to remove the connector and lower-quality reads, and thus, only high-quality clean reads were retained. The edgeR software (v3.16.5) was employed to normalized the data, and differentially expressed mRNAs were analyzed.

### Bioinformatics analysis

Gene ontology (GO) and Kyoto encyclopedia of genes and genomes (KEGG) analysis were conducted for the target differentially expressed mRNAs using DAVID (Database for Annotation, Visualization, and Integrated Discovery).

### US/PA dual modal imaging

HPDC NPs at different concentrations were used for both PA and US imaging in vitro. The PA images and corresponding PA values were collected using the PA imaging system. The US imaging and corresponding values were detected using the US diagnostic instrument (Esaote, Italy). The in vitro phase transition behavior of PFH was studied before and after exposing PDC NPs and HPDC NPs to NIR − I laser (1 W cm^−2^, 5 min). For US/PA imaging in vivo, female rats with lymph nodes metastasis were subcutaneously injected with 50 μL of PDC NPs (2 mg mL^−1^) and HPDC NPs (2 mg mL^−1^) into the mammary gland, respectively. The PA images and corresponding PA values of metastatic lymph nodes were recorded at predetermined time intervals (5, 15, 30, 60, and 120 min) post-injection. For in vivo US imaging, the metastatic lymph nodes were exposed to NIR − I laser irradiation for 5 min (the power intensity was set as 1.5 W cm^−2^) at 120 min after subcutaneously injection.

### In vivo biocompatibility evaluation of HPDC NPs

Female rats (n = 5 in each group) were subcutaneously injected with PBS (50 μL, denoted as control) and HPDC NPs (50 μL, 2 mg mL^−1^). Blood and main organs were collected at 1, 7, and 14 days after injection for routine blood examination, serum biochemical analysis, and histological analysis by hematoxylin and eosin (H&E) staining.

Female rats (n = 5 in each group) were subcutaneously injected with PBS (50 μL), HPDC NPs (2 mg mL^−1^, 50 μL), or DOC suspension (1.6 mg mL^−1^, 50 μL), respectively. The skin tissue around the injection site was sampled at 2 and 24 h after injection for histological analysis by H&E staining.

### In vivo photothermal performance of HPDC NPs

Female rats were subcutaneously injected with 50 μL of PBS, CNPs, and HPDC NPs (2 mg mL^−1^) into the mammary gland metastatic lymph nodes. The metastatic lymph nodes of the rats were then exposed to 808 nm laser irradiation (1.5 W cm^−2^, 5 min). Additionally, variations in the temperature at metastatic lymph nodes sites were observed by using an IR thermal image recorder.

### Synergistic chemo-photothermal therapy in vivo

Thirty female rats with lymph nodes metastasis were bred at the Laboratory Animal Center of Chongqing Medical University. Walker256 cancer cells at a density of 1 × 10^6^ cells per rat were suspended in 100 μL of PBS and subcutaneously injected into the mammary gland to establish the lymph node metastasis model. After 2 weeks, the rats were randomly divided into six groups (n = 5 per group) as follows: control, 808 nm laser only, DOC only, HPDC NPs only, HPC NPs + 808 nm laser, and HPDC NPs + 808 nm laser groups. After 2 h of subcutaneously injection, the rats were irradiated with 808 nm laser (1.5 W cm^−2^, 5 min). Next, the rats were killed, and the metastatic lymph nodes were dissected and subjected to H&E, TdT-mediated dUTP Nick-End Labeling (TUNEL), and Ki-67 antibody staining for histological analysis.

### Statistical analysis

The data are represented as the mean ± standard deviation, and significant differences between two groups were analyzed using Student’s two-tailed *t* test (*p < 0.05, **p < 0.01, ***p < 0.001, ****p < 0.0001).

## Results and discussion

### Characterization and in vitro photothermal properties of HPDC NPs

**Fig. 1:**
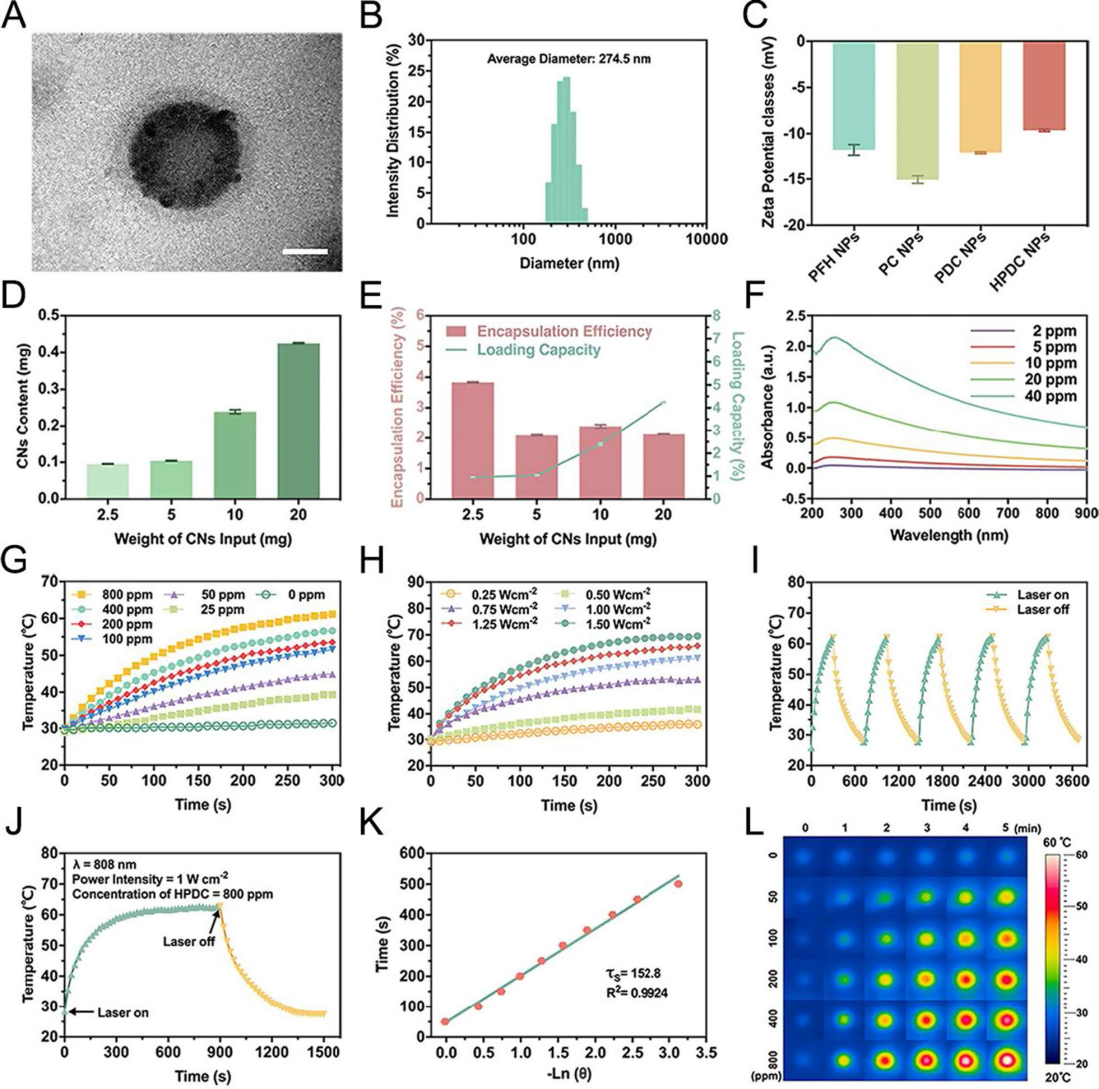
Characterization and in vitro photothermal performance of HPDC NPs. **A** TEM image of HPDC NPs (Scale bar: 100 nm). **B** Size distribution of HPDC NPs measured by DLS. **C** Zeta potential measurements of PFH NPs, PC NPs, PDC NPs, and HPDC NPs. **D** CNs content and **E** the DEE and DLC of NPs with different initial CN loading (2.5, 5, 10, and 20 mg) (n = 3 per group). **F** Ultraviolet absorption curves of varying concentrations of CNs (2, 5, 10, 20, and 40 ppm). **G** Heating curves of varying concentrations of HPDC NPs upon 808 nm laser irradiation (1 W cm^−2^ for 5 min). **H** Temperature curves of HPDC NPs aqueous dispersion exposed to 808 nm laser irradiation at varied power densities. **I** Temperature curves of heating and cooling processes of HPDC NPs aqueous dispersion over five cycles. **J** Photothermal conversion efficiency of HPDC NPs aqueous dispersion exposed to 808 nm laser irradiation (1 W cm^−2^ for 5 min). **K** Thermal time constant of HPDC NPs obtained from the cooling period. **L** IR thermal images of different concentrations of HPDC NPs dispersion upon 808 nm laser irradiation (1 W cm^−2^ for 5 min)

PDC NPs were synthesized by an improved three-step emulsion approach. HPDC NPs were fabricated by the carbodiimide technique (Scheme 1A). The morphology and size distribution of HPDC NPs were studied by TEM (Fig. 1A). The TEM image revealed the spherical morphology, PLGA shell, the middle layer of CNs, and the PFH core structure of HPDC NPs with an average diameter of about 260 nm. Furthermore, the average hydrodynamic particle size of HPDC NPs was measured to be 274.5 nm through DLS measurement (Fig. 1B), which was consistent with the TEM observed results. Since the gaps between lymphatic endothelial cells were much larger than those of vascular endothelial cells, these large-sized NPs could only enter into the lymphatic system but not the blood circulation system after subcutaneous injection. Before loading of PFH and DOC, the size distribution and morphology of CNs-PLGA NPs (denoted as CNP NPs) were revealed through DLS and TEM measurements (Additional file 1: Figs. S1 and S2), which showed that the CNP NPs have an average diameter around 236.7 nm and spherical structure. Before modification with anti-HIF 1α antibody, the average hydrodynamic diameter of PDC NPs was determined to be 263.8 nm (Additional file 1: Fig. S3). The zeta potential of PFH NPs, PFH-CNs NPs, PDC NPs and HPDC NPs was detected to be − 11.87 ± 0.57, − 15.07 ± 0.42, − 12.17 ± 0.12, and − 9.65 ± 0.12 mV, respectively (Fig. 1C). The encapsulation occurred due to the negatively charged CNs and positively charged DOC and anti-HIF 1α antibody. The loading effects of various initial CNs on the practical CNs content (Fig. 1D) and the DEE and DLC (Fig. 1E) of HPDC NPs were evaluated based on the ultraviolet–visible (UV–vis)-NIR spectra. An increase in the input of CNs also increased the content of CNs in HPDC NPs (CN content in HPDC NPs prepared with 2.5, 5, 10, and 20 mg of CNs were 95.81 ± 0.46, 104.44 ± 0.96, 239.37 ± 5.26, and 424.99 ± 1.87 μg, respectively). Similarly, the loading capacity (DLC of CNs in HPDC NPs prepared with 2.5, 5, 10, and 20 mg of CNs were 0.96 ± 0.46, 1.04 ± 0.96, 2.39 ± 5.26, and 4.25 ± 1.87%, respectively) raised with the increased of CNs input amount, whereas the encapsulation efficiency (DEE of CNs in HPDC NPs prepared with 2.5, 5, 10, and 20 mg of CNs were 3.83 ± 0.02, 2.09 ± 0.02, 2.39 ± 0.05, and 2.12 ± 0.01%, respectively) did not exhibit any such trend. Based on these results, we selected the initial CNs amount of 10 mg for subsequent experiments on the premise of loading as much CNs as possible and not wasting raw materials.

As exhibited in Fig. 1F, the UV–vis-NIR absorbance values of CNs at 808 nm increased with the incremental amounts of CNs, and the calculated mean extinction coefficient was 21.64 L g^−1^ cm^−1^ (Additional file 1: Fig. S4), which revealed that HPDC NPs could display a photothermal effect with NIR − I laser. The photothermal conversion efficiency was examined by irradiating various compositions of HPDC NPs solution, and the results showed that the temperature rise curves were related to the corresponding concentrations of HPDC NPs, laser power, and irradiation time (Fig. 1G and H). The temperature of 800 ppm HPDC NPs rose from 28.8 to 61.1 °C after 5 min of NIR − I laser exposure (Fig. 1G and L). As can be seen from Fig. 1H, the temperature of HPDC NPs aqueous dispersion increased from 41.7 °C to 69.5 °C by managing the laser power density from 0.5 W cm^−2^ to 1.5 W cm^−2^, which proved that the photothermal conversion property depended on the power density of NIR − I laser. In addition, there was no obvious variation in the photothermal properties of HPDC NPs after five heating and cooling cycles under 808 nm laser irradiation, proving that HPDC NPs have satisfactory photothermal stability (Fig. 1I). More importantly, the calculated photothermal conversion efficiency of HPDC NPs was 28.9% (Figs. 1J and K), which was even better than that of many photothermal conversion nanoagents, including HCuSNPs (23.8%) [51], AIPH@Nb2C@mSiO2 NPs (27.03%) [52], and living photosynthetic bacteria (PSB) (27.3%) [53]. These results indicated that HPDC NPs could be used as a potential photothermal agent for future photothermal therapy attributing to its prominent photothermal conversion performance.

### Drug loading and release performance of HPDC NPs

**Fig. 2:**
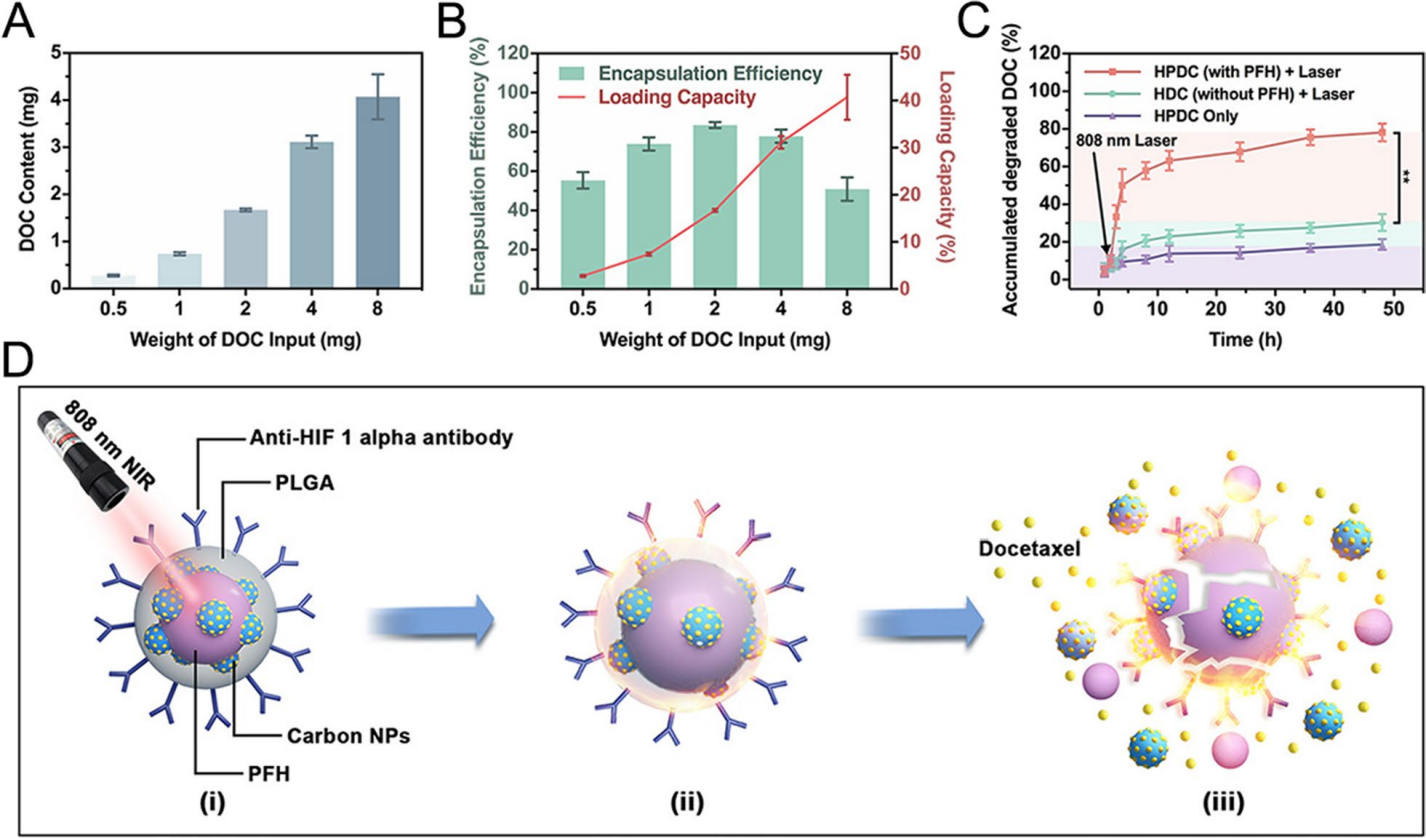
Drug release performance of HPDC NPs. **A** DOC content and **B** the DEE and DLC of NPs with varied initial input weights (0.5, 1, 2, 4, and 8 mg) (n = 3 per group). **C** The DOC release status of various treatment groups over 48 h, including HPDC only, HDC + NIR − I laser and HPDC + NIR − I laser groups. The NPs were exposed to laser irradiation (1 W cm^−2^, 5 min) at 2 h (n = 3 per group). **D** Schematic diagram of laser-triggered release behavior of DOC from HPDC NPs

To verify the capability of NIR − I laser irradiation to control drug release, the drug-loading effects of varying original DOC amounts on the practical DOC content, DLC, and DEE were analyzed using HPLC (Additional file 1: Fig. S5). With an increase in the initial DOC input, the DOC contained in HPDC NPs raised correspondingly (DOC content in HPDC NPs prepared with 0.5, 1, 2, 4, and 8 mg of DOC was 0.27 ± 0.02, 0.73 ± 0.03, 1.67 ± 0.02, and 3.11 ± 0.13 mg, respectively) (Fig. 2A). Similarly, the DLC (DLC of DOC in HPDC NPs prepared with 0.5, 1, 2, 4, and 8 mg of DOC was 2.76 ± 0.21, 7.39 ± 0.33, 16.71 ± 0.30, 31.16 ± 1.34, and 40.72 ± 4.77%, respectively) also elevated with an increase in the initial DOC input. In contrast, the DEE of DOC in HPDC NPs increased gradually as the initial DOC input raised from 0.5 to 2 mg and then decreased (Fig. 2B). Based on the experimental results above, the best DEE (83.54%) and better DLC (16.71%) were obtained when 2 mg of DOC was used as the initial input.

Microscopic images of HPDC NPs revealed that the number of microbubbles raised gradually after NIR − I laser exposure (Additional file 1: Fig. S6), laser irradiation induced a phase transition due to the vaporization of the liquid PFH core, controlling the drug release. The DOC release from HPDC NPs was investigated by comparing the release in three groups: HPDC only, HDC + 808 nm laser, and HPDC + 808 nm laser. As shown in Fig. 2C, after the drug release began, the NPs were irradiated with an 808 nm infrared laser (1 W cm^−2^ for 5 min) in the second hour. No significant change was observed in the HPDC only and HDC + 808 nm laser groups, while explosive drug release was observed in the HPDC + 808 nm laser group. Therefore, it could be inferred that the remarkably enhanced release of DOC was due to the rupture of HPDC NPs caused by the vaporization of PFH induced by laser irradiation (Fig. 2D).

### In vitro targeting behavior of HPDC NPs

**Fig. 3:**
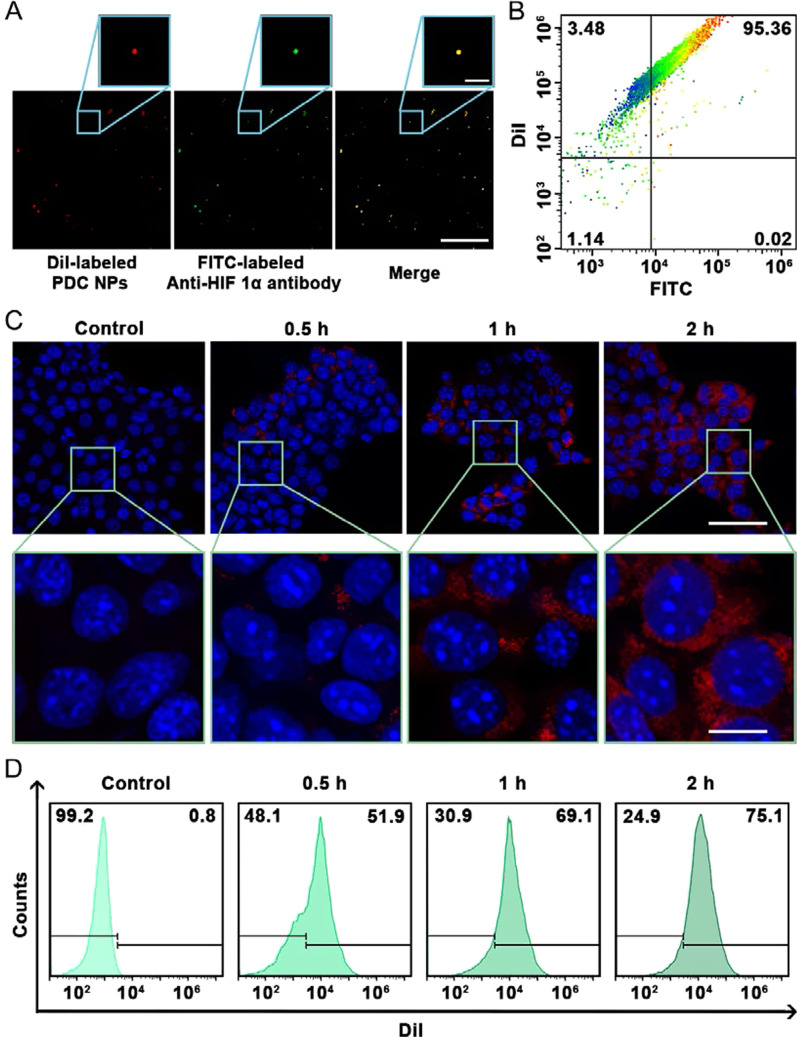
Cellular targeting performance of HPDC NPs. **A** CLSM images of DiI-labeled PDC NPs co-incubated with FITC-labeled anti-HIF 1α antibody for 2 h. NPs bound with anti-HIF 1α antibody exhibited yellow fluorescence in the merged images (magnification in the small images was 1800 ×). **B** Flow cytometry analysis of DiI-labeled PDC NPs and FITC labeled excess antibody after incubation for 2 h through covalent coupling reaction. **C** CLSM analysis (Scale bars were 100 and 20 μm, respectively) and **D** flow cytometry analysis of cellular endocytosis of DiI-labeled HPDC NPs (100 ppm) in walker256 tumor cells after varied incubation durations

NPs with effective targeting behavior are important to achieve precision therapy. The binding of PDC NPs with anti-HIF 1α antibody was detected by CLSM. The overlap (yellow fluorescence) of DiI-labeled PDC NPs (red fluorescence) and FITC-labeled anti-HIF 1α antibody (green fluorescence) demonstrated the successful binding between PDC NPs and anti-HIF 1α antibody (Fig. 3A). The binding efficiency of PDC NPs and excess anti-HIF 1α antibody was 95.36 ± 2.46%, which further quantitatively confirmed that the anti-HIF 1α antibody was successfully attached to PDC NPs (Fig. 3B). Depending on the binding efficiency of excess PDC NPs and anti-HIF 1α antibody (68.25 ± 6.93%) (Additional file 1: Fig. S7), the molar ratio of PDC NPs and anti-HIF 1α antibody was calculated to be about 110. In addition, the targeting behavior of HPDC NPs to Walker256 cells was investigated by CLSM. As shown in Fig. 3C, the red fluorescence intensity of DiI-labeled HPDC NPs in cancer cells increased with the prolongation of incubation time (0–2 h), indicating that the HPDC NPs were effectively phagocytosed over time. Moreover, the red fluorescence of DiI-labeled HPDC NPs in tumor cells appeared round the nucleus, indicating that HPDC NPs presents nucleophilic targeting due to anti-HIF 1α antibody. In addition, the endocytosis fluorescence intensity of DiI-labeled HPDC NPs in Walker256 cells was investigated by flow cytometry (Fig. 3D). The intensity of red fluorescence in tumor cells increased with time. The fluorescence intensity in the HPDC NPs group at 2 h was 96.22-fold higher than that in the control group (Additional file 1: Fig. S8). The results further confirmed that the effectively endocytosis of HPDC NPs, and the content of HPDC NPs in the tumor cells increased with the culture duration, consistent with CLSM observations. These properties make HPDC NPs a good candidate to achieve precisely controlled drug release and photothermal therapy.

### In vitro biocompatibility and synergy therapy

**Fig. 4:**
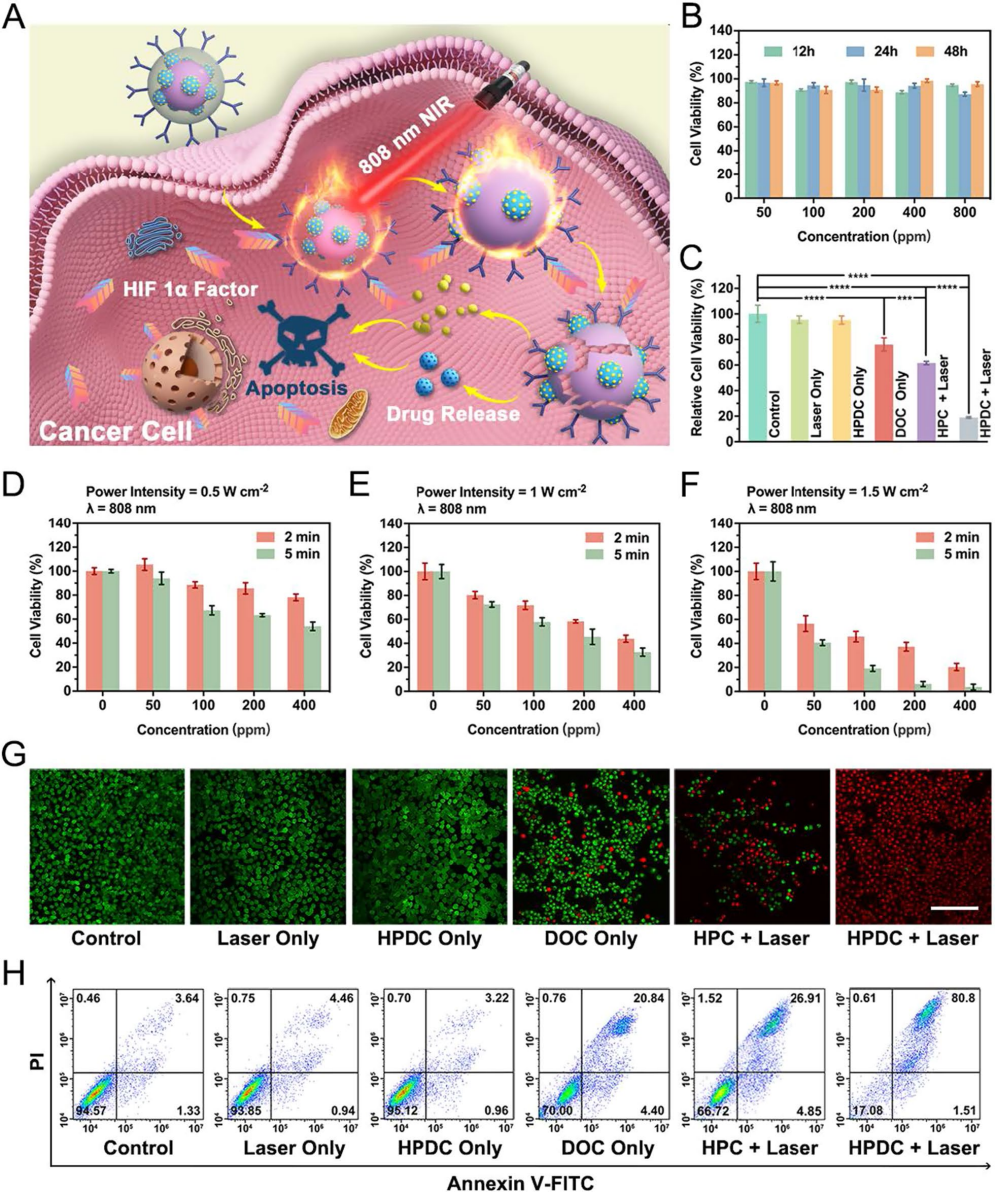
In vitro synergistic photonic hyperthermia and chemotherapy. **A** Schematic illustration of HPDC NPs for synergistic hyperthermia and chemotherapy against walker256 tumor cells. **B** Relative cell viability of walker256 cells co-incubated with HPDC NPs at varied concentrations for different treatment durations. **C** Relative cell viability after varied treatments, including control, NIR − I laser only, HPDC NPs only, DOC only, HPC NPs + NIR − I laser, and HPDC NPs + NIR − I laser groups. All data are presented as mean ± standard and Student’s two-tailed *t* test (*p < 0.05, **p < 0.01, ***p < 0.001, ****p < 0.0001), n = 3 per group. **(D–F)** Relative cell viability of HPDC NPs at different concentrations (0, 50, 100, 200, and 400 ppm) under 808 nm laser irradiation at the power intensity of 0.5, 1, and 1.5 W cm^−2^ for 2 and 5 min. **G** Observed CLSM images of walker256 cells stained by PI and calcein-AM (Scale bar was 100 μm). **H** Flow cytometry apoptosis assay of cancer cells stained by Annexin-FITC and PI after varied treatments: control, laser only, HPDC NPs only (200 ppm), DOC only (35 ppm), HPC NPs (200 ppm) + NIR − I laser, and HPDC NPs (200 ppm) + NIR − I laser groups. The power density was 1.0 W cm^−2^ and the irradiation time was 5 min

The in vitro hyperthermia-combined chemotherapy of HPDC NPs was determined based on cell survival in the DOC, HPC NPs, and HPDC NPs groups in the absence and presence of NIR − I laser irradiation assessed by the standard CCK-8 assay (Fig. 4A). The in vitro biological safety of HPDC NPs was also analyzed by measuring the viability of cells incubated with different concentrations of HPDC NPs by the standard CCK-8 assay (Fig. 4B). No significant cytotoxicity towards walker256 cells was detected after incubation with HPDC NPs, even at 800 ppm concentration and 48 h incubation duration. Then, the in vitro efficiency of synergistic photothermal and chemotherapy was tested via CCK-8 assay. As the concentration of HPDC NPs and power density of NIR − I laser increased, the cytotoxicity of HPDC NPs exposed to NIR − I laser was much higher than that of HPDC NPs alone (Fig. 4C–F). In the presence of 808 nm laser irradiation (1 W cm^−2^, 5 min), only 19.03% of cells survived after treatment with 200 ppm HPDC NPs, while 76.13% and 61.55% of tumor cells survived after treatment with 35 ppm DOC alone and 200 ppm HPC NPs + laser exposure, respectively (Fig. 4C). These results demonstrated that HPDC NPs under laser irradiation could effectively induce tumor cell apoptosis through photothermal therapy combined with their ability to release the chemotherapeutic drug in situ.

The synergistic efficacy of photothermal therapy and chemotherapy was also confirmed by CLSM (Fig. 4G), where calcein-AM (green fluorescence) and PI (red fluorescence) stained the live and dead cells, respectively. The control group, laser only group and HPDC NPs only group exhibited a strong green fluorescence and almost no red fluorescence, indicating that laser irradiation and HPDC NPs did not significantly damage the Walker256 cells. However, the HPDC NPs + NIR − I laser group showed remarkable red fluorescence, while the DOC group and HPC + NIR − I laser group revealed both green and red fluorescence, indicating that the combination of photothermal therapy and chemotherapy resulted in effective tumor cell killing. Additionally, the samples were stained with Annexin V-FITC and PI before flow cytometry analysis. After laser irradiation, the cellular apoptosis rate induced by HPDC NPs was 82.31%, which was significantly higher than that produced by DOC (25.24%) and HPC NPs (31.76%) under identical experimental conditions (Fig. 4H and Additional file 1: Fig. S9). The apoptosis rate of incubation with HPDC NPs alone was just 4.16%. These results not only further certified that photothermal therapy combined with in situ chemotherapy prompted significant tumor cell death through early and late apoptotic pathways but also confirmed the cytocompatibility of HPDC NPs.

### Mechanistic analysis of HPDC NPs in hyperthermia-combined chemotherapy

**Fig. 5:**
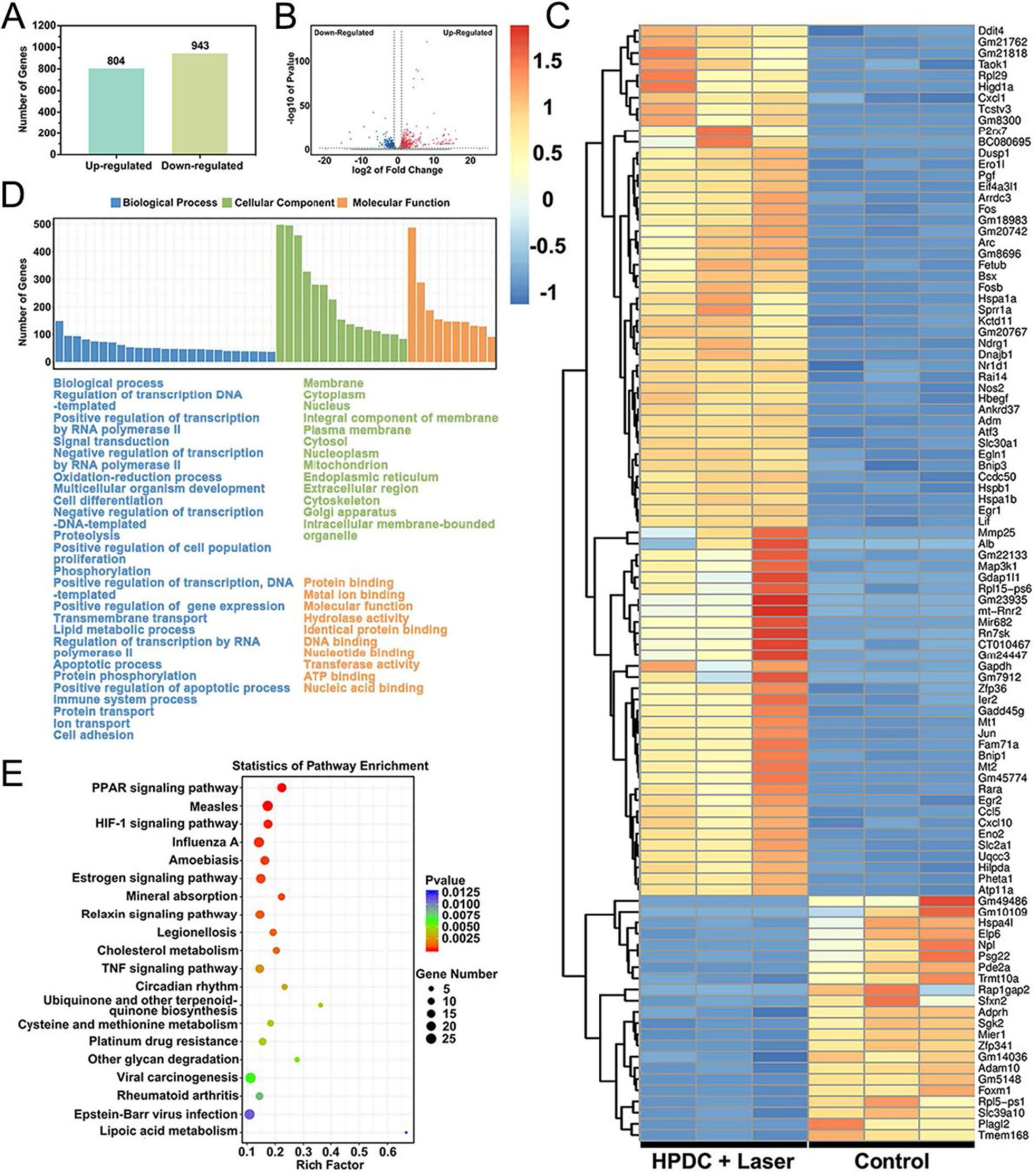
Mechanistic role of HPDC NPs in the synergistic hyperthermia and chemotherapy. **A** The number of SDEGs between control and HPDC NPs + 808 nm laser irradiation groups. **B** The volcano plot of SDEGs between the control and HPDC NPs + NIR − I laser irradiation samples. Red dots represent upregulated genes and blue dots represent downregulation genes. **C** Heatmap of the SDEGs associated with cell cycle and apoptosis. Warm orange color indicates the significantly upregulated genes and cold blue color represents the remarkably downregulated genes (fold change (FC) ≥ 2.0 (or − 2.0) and *P* < 0.05). **D** GO and **E** KEGG enrichment pathway analysis of the SDEGs

The potential therapeutic mechanisms of HPDC NPs exposed to NIR − I laser irradiation were studied by investigating the mRNA profiles of Walker256 tumor cells exposed to PBS (denoted as the control group) or HPDC NPs + 808 nm laser irradiation (denoted as treatment group). RNA sequencing revealed 1747 significantly differentially expressed genes (SDEGs) between the control and treatment groups, including 804 upregulated SDEGs and 943 downregulated SDEGs (Fig. 5A). A volcano plot based on these SDEGs demonstrated differentially dysregulated genes, suggesting that the genes exhibited significant differences in their expression between the control and HPDC + 808 nm laser groups (Fig. 5B). Next, genes related to cellular apoptosis were visualized in the heatmap after Walker256 cells were incubated with HPDC NPs and irradiated by 808 nm laser subsequently (Fig. 5C). Among 100 genes associated with apoptosis, 22 genes were downregulated and 78 upregulated. The overexpression of fetuin beta (FetuB) induces the suppression of tumor growth [54], and the downregulation of phosphodiesterase 2A (Pde2a) could inhibit thrombin-mediated redox-sensitive endothelial cell proliferation and angiogenesis via Rac1 and NADPH oxidase 2 [55]. Furthermore, GO enrichment analysis, including biological process (BP), cellular component (CC), and molecular function (MF), revealed that the cells responded to the hypoxic tumor microenvironment through metabolic adaptation at both the cell and organism levels, which might have facilitated the targeting behavior of anti-HIF 1α antibody (Fig. 5D and Additional file 1: Fig. S10). KEGG pathway enrichment analysis was conducted to further study the mechanism by which HPDC NPs acted on cancer cells by synergistic chemotherapy and photonic hyperthermia. As shown in Fig. 5E, the PPAR signaling pathway and HIF-1 signaling pathway were significantly enriched among the top 20 enriched pathways. The PPAR signaling pathway not only involves regulation of lipid metabolism, adipogenesis, maintenance of metabolic homeostasis, and expression of inflammatory genes but also induces anticancer effects in several forms of cancer (Additional file 1: Fig. S11) [56]. The enriched HIF-1 signaling pathway that mediated adaptive response to the reduced oxygen availability confirmed the specificity and effectiveness of the targeting behavior of anti-HIF 1α antibody [57].

### US/PA dual modal imaging in vitro and in vivo

**Fig. 6:**
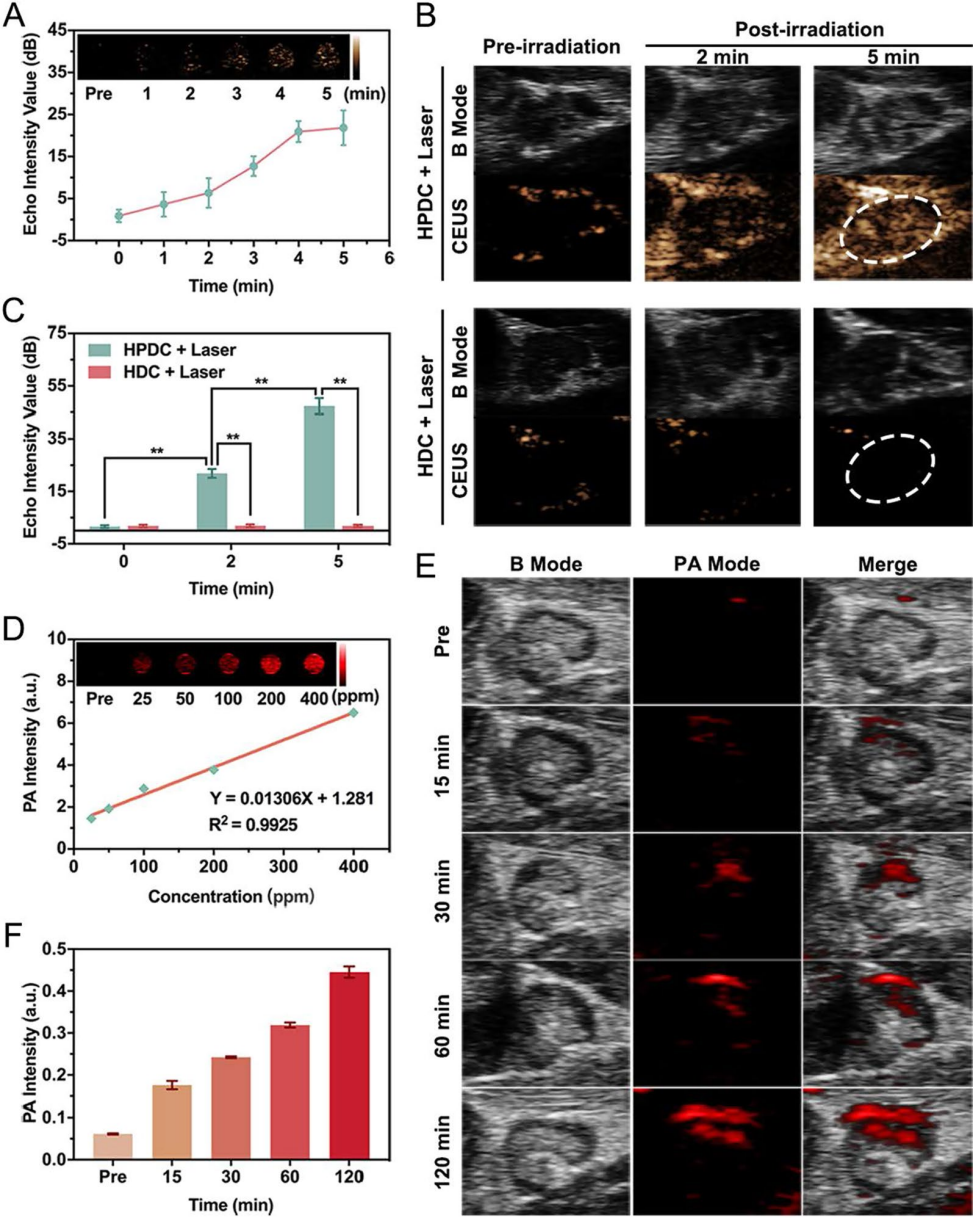
PA/US imaging of HPDC NPs in vitro and in vivo. **A** In vitro US images in CEUS of HPDC NPs at varying radiation times (pre-irradiation and 1 to 5 min post-irradiation) and the corresponding echo intensity values (1 W cm^−2^). **B** In vivo US images in CEUS of lymph nodes after the subcutaneous injection of HPDC pre- and post-irradiation (1.5 W cm^−2^ for 2 and 5 min). **C** The in vivo echo intensity values in CEUS. Data are presented as mean ± standard and Student’s two-tailed *t* test (*p < 0.05, **p < 0.01) (n = 3 per group). **D** In vitro PA images and the fitted curves between PA values with varying concentrations of HPDC NPs (25, 50, 100, 200, and 400 ppm). **E** PA images in metastatic lymph nodes after the subcutaneous injection of HPDC NPs at different times. **F** PA signal intensity values at lymph nodes at varied intervals post-injection

The accumulation of HPDC NPs in metastatic lymph node sites was detected by US and PA-guided imaging before in vivo synergistic photonic hyperthermia and chemotherapy. Given the presence of PFH in HPDC NPs and the excellent optical droplet vaporization performance of PFH, we evaluated the US imaging capability of HPDC NPs in vitro and in vivo. As displayed in Fig. 6A, the echo intensity of US imaging in contrast-enhanced ultrasound (CEUS) was elevated with an extension in the laser irradiation duration (1–5 min). After 5 min of 808 nm laser exposed, the echo intensity of HPDC NPs increased markedly from 0.91 dB to 21.84 dB, but the increase was weakly from 20.93 dB to 21.84 dB at the last minute, demonstrating that HPDC NPs represented excellent US imaging capability after laser irradiation for 5 min. The in vivo US imaging capability of HPDC NPs is exhibited in Fig. 6B and C. Compared with the PDC NPs group that presented almost no US imaging ability, the HPDC NPs group showed outstanding echo signal as high as 47.31 dB in CEUS after laser irradiation. The results above indicated that HPDC NPs are an ideal US contrast agent with remarkable gas microbubble generation in response to NIR − I light irradiation. Importantly, US imaging could also monitor the morphologic changes of tumor tissues during the whole treatment period in real-time [58], which could assist subsequent treatment and compensate for the deficiency of PA imaging to some extent.

Due to the prominent photothermal conversion performance of HPDC NPs, in vitro and in vivo PA images were acquired using a Vevo LAZR PA imaging system. As a desirable PA imaging agent, HPDC NPs exhibited excellent PA imaging contrast enhancement in vitro, and the PA signal values enhanced linearly with increased concentrations from 25 to 400 ppm (Fig. 6D), allowing PA imaging-guided targeted tumor treatment. Then, HPDC NPs were injected subcutaneously around the mammary gland, and in vivo PA imaging of metastatic lymph nodes was conducted. The PA signal images at lymph node sites were detected at varied time intervals after the subcutaneous injection of HPDC NPs (Fig. 6E) and PDC NPs (Additional file 1: Fig. S12A). The PA signal intensity at the lymph node region reached the maximum 2 h after the injection of HPDC NPs with targeting behavior (Fig. 6F), while the PA signal intensity rapidly reached the maximum 0.5 h after the injection of PDC NPs and then began to decline (Additional file 1: Fig. S12B). The in vivo PA imaging results confirmed that the HPDC NPs could not only accumulate in metastatic lymph nodes through their passive targeting behavior but also had a sufficient residence time in the lymph node region via intracellular targeting at HIF 1α factor due to the anti-HIF 1α antibody modification. Due to the effective accumulation and residence time of HPDC NPs in metastatic lymph nodes, the development time of PA imaging was longer than that of one-time development of US imaging, which was conducive to photothermal therapy with laser irradiation for multiple times [59, 60].

### In vivo biosafety assay

**Fig. 7:**
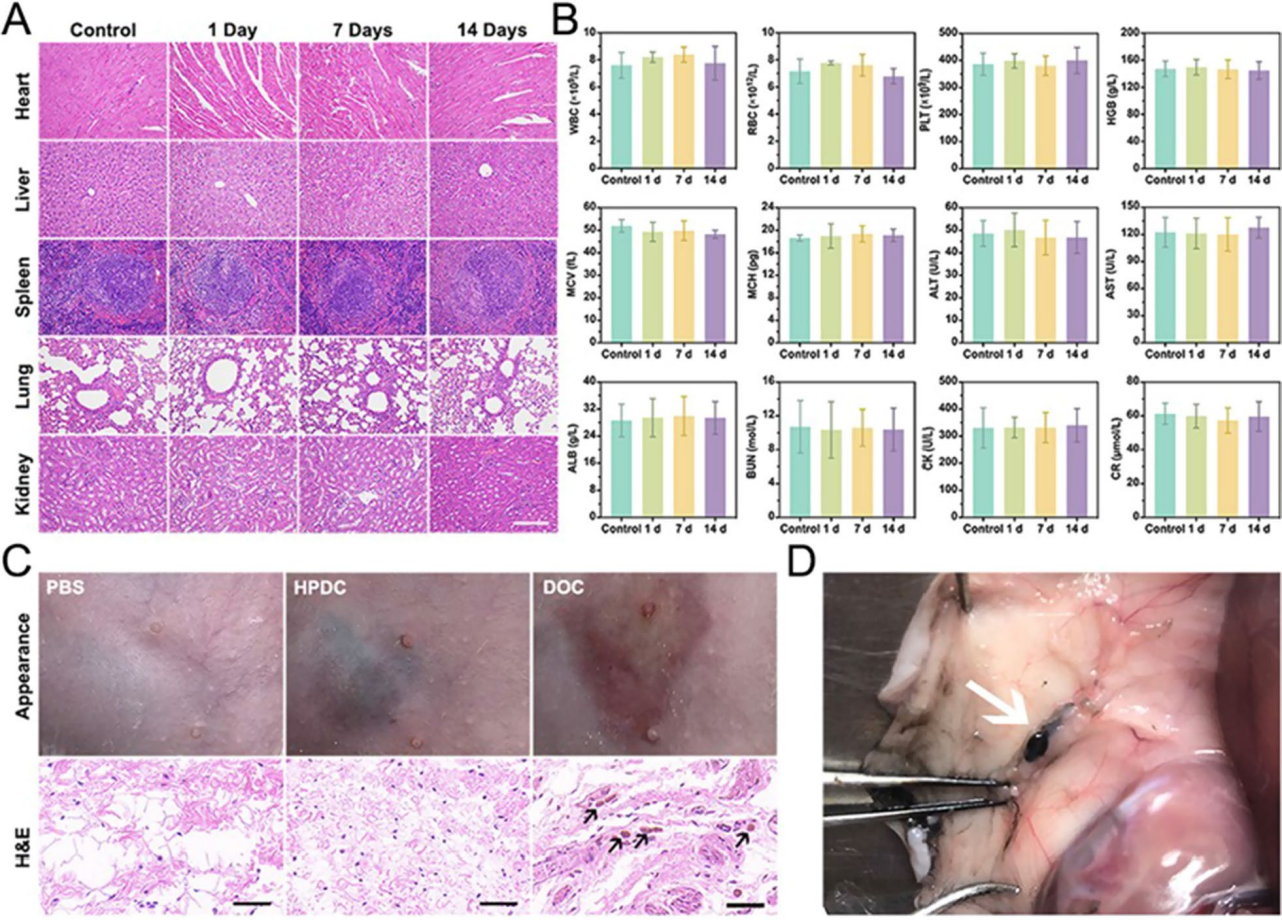
In vivo biosafety evaluation and anatomical location of HPDC NPs. **A** H&E stained images acquired from the major organs (heart, liver, spleen, lung, and kidney) of female rats after the subcutaneous injection with PBS (control) and HPDC NPs (1, 7, and 14 days) (Scale bars: 200 μm). **B** Routine blood parameters and biochemical indexes examination of the female rats after subcutaneous injection with PBS (control) and HPDC NPs for 1, 7, and 14 days feeding, including white blood cell (WBC), red blood cell (RBC), platelet (PLT), hemoglobin (HGB), mean corpuscular volume (MCV), mean corpuscular hemoglobin (MCH), alanine aminotransferase (ALT), aspartate aminotransferase (AST), albumin (ALB), blood urea nitrogen (BUN), creatine kinase (CK), and creatinine (CR) (n = 3 per group). **C** Appearance and H&E stained images of the skin 2 h after the subcutaneous injection of PBS, HPDC NPs and DOC (Scale bars: 50 μm). **D** Anatomical location of lymph nodes after the subcutaneous injection of HPDC NPs

Systematically, the in vivo potential toxicity of HPDC NPs was synthetically assessed in female rats to demonstrated its biosafety and potential clinical translation. H&E staining of major organs in rats receiving a subcutaneous injection of HPDC NPs presented almost no histopathological lesions (Fig. 7A). Blood routine analysis and blood biochemical tests also exhibited no toxicity related to HPDC NPs (Fig. 7B). More importantly, the appearance and H&E staining of the skin at the injection site showed minor skin damage and pathological alteration after injecting PBS and HPDC NPs, whereas obvious tissue damage was found in the DOC group after 2 h (Fig. 7C). After 24 h of observation, no obvious tissue damage was observed in the PBS and HPDC NPs groups (Additional file 1: Fig. S13). In addition, HPDC NPs still presented lymphatic tropism and dyeing ability to the same extent as CNs after subcutaneous injection (Fig. 7D). In some cases where surgery is necessary, stained lymph nodes make it easier for surgeons to locate and remove the metastatic lymph nodes, avoiding injury to the axillary lymphatic system due to the otherwise aimless total resection. Thus, the results of the present study demonstrate the biological safety and potential clinical translation of HPDC NPs, and it was further certified that chemotherapeutic drugs can be effectively protected by PLGA encapsulation to reduce or even avoid leakage.

### In vivo anti-tumor efficiency of HPDC NPs

**Fig. 8:**
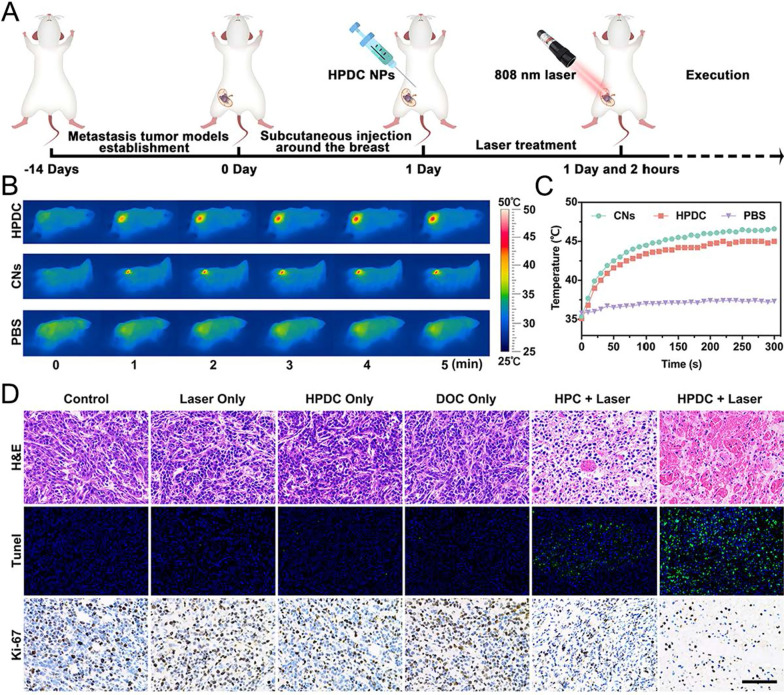
In vivo synergistic photonic hyperthermia and chemotherapy. **A** Schematic diagram of the establishment and treatment of metastatic tumor model rats (n = 5 in each group). **B** Photothermal images and **C** temperature curves of lymph nodes at varying intervals after the subcutaneous injection of PBS, CNs, or HPDC NPs upon NIR − I laser irradiation (1.5 W cm^−2^ for 5 min). **D** H&E, TUNEL, and Ki-67 antibody staining results of metastatic lymph nodes in the treated groups (Scale bars: 50 μm)

The in vivo efficacy of synergistic photonic hyperthermia and chemotherapy was evaluated in rats with lymph node metastasis established by injecting Walker256 cells and randomly divided into six groups: control, laser only, HPDC NPs only, DOC only, HPC NPs + 808 nm laser, and HPDC NPs + 808 nm laser groups. The metastatic lymph nodes were irradiated with an 808 nm laser for 5 min 2 h after subcutaneous injection (Fig. 8A). The increase in temperature at the lymph node region in different treatment groups was monitored by using an IR thermal imaging camera. After irradiation by 808 nm laser for 5 min, the temperature at lymph node site in HPC NPs + 808 nm and HPDC NPs + 808 nm groups reached 46.6 °C and 45.0 °C, respectively, whereas it was only 1.5 °C increased in the PBS group (Fig. 8B and C). Furthermore, the therapeutic effects after varied treatments were performed by H&E, TUNEL, and Ki-67 antibody staining analysis. Prominent apoptosis/necrosis of lymph node tissues was found in the HPC + 808 nm laser and HPDC + 808 nm laser groups. Moreover, the level of cell apoptosis and necrosis in the HPDC + NIR − I laser group was remarkably higher than that in the other treatment groups (Fig. 8D). In conclusion, in situ photothermal therapy-combined chemotherapy presented optimally suppressed metastatic lymph node tumors and could even replace conventional chemotherapy and radiation therapy.

## Conclusions

A visualized precision medicine nanoplatform of metastatic lymph nodes via lymphatic delivery has been successfully established to achieve US/PA imaging-guided and laser-activated hyperthermia-combined DOC-based chemotherapy. Under the monitoring by real-time US imaging and repeatable PA imaging, HPDC NPs effectively carried both CNs and DOC into the metastatic lymph nodes due to their targeting behavior, where nanoparticles could be broken upon NIR − I laser irradiation and subsequently release DOC molecules. The constructed nanoplatform could induce CNs-mediated tumor photothermal therapy with a photothermal-conversion efficiency reached as high as 28.9%. Momentously, the biosafety of initial materials, preparation methods, and subcutaneous injection of nanoparticles was also demonstrated. The targeted effects of HPDC NPs in the metastatic lymph nodes were confirmed both in vitro and in vivo. This study not only provides a laser-triggered highly efficient in situ chemotherapy nanosystem for the targeted chemo-hyperthermia synergistic treatment of metastatic lymph nodes but also represents an alternative delivery route through the lymphatic system that avoids additional injury caused by drugs entering the blood circulation.

## Supplementary Information

**Additional file 1: Figure S1.** TEM image of CNs-PLGA NPs (denoted as CNP NPs). **Figure S2.** Size distribution of CNP NPs as measured by DLS. **Figure S3.** Size distribution of PDC NPs as measured by DLS. **Figure S4.** The fitting curve of extinction coefficient of CNs at 808 nm. **Figure S5.** The standard curve of DOC measured by HPLC. **Figure S6.** In vitro NIR-I controlled phase transition of HPDC NPs. Microscopic images of HPDC NPs at different times irradiation, including **A** pre-irradiation, **B** 2 min post-irradiation, and **C** 5 min post-irradiation (1 W cm^−2^). **Figure S7.** Flow cytometry analysis of DiI-labeled excess PDC NPs and FITC labeled antibody after incubation for 2 h through covalent coupling reaction. **Figure S8.** Fluorescence intensity of DiI-labeled HPDC NPs (100 ppm) in Walker256 cancer cells after different incubation durations (n = 3 per group). **Figure S9.** The percentage of live, early apoptosis and late apoptosis cells in different treatment groups. (1: control (treated with PBS), 2: Laser only, 3: HPDC only, 4: DOC only, 5: HPC + 808 nm laser, and 6: HPDC + 808 nm laser groups). **Figure S10.** GO enrichment analysis of SDEGs. **Figure S11.** The pathway map of PPAR signaling pathway. Yellow marked nodes are associated with up-regulated enriched genes, blue marked nodes are associated with down-regulated enriched genes, and green nodes have no significance. **Figure S12. A** In vivo PA images in lymph tissues after subcutaneous injection of PDC NPs at varying time intervals. **B** PA signal intensity values at lymph regions after varied treatment durations (n = 3 per group). **Figure S13.** Skin appearance and H&E stained images after 24 h of subcutaneous injection with PBS, HPDC NPs and DOC, respectively (Scale bars: 50 μm).

## Data Availability

The data are available in the main manuscript, Additional information files are available from the corresponding author by request.
