# Supplementary material for: A novel targeted multifunctional nanoplatform for visual chemo-hyperthermia synergy therapy on metastatic lymph nodes via lymphatic delivery
Source: J Nanobiotechnology. 2021 Dec 20;19:432. doi: 10.1186/s12951-021-01186-8 (PMC8686382; doi:10.1186/s12951-021-01186-8)
Supplement: Supplementary file 1 — Additional file 1: Figure S1. TEM image of CNs-PLGA NPs (denoted as CNP NPs). Figure S2. Size distribution of CNP NPs as measured by DLS. Figure S3. Size distribution of PDC NPs as measured by DLS. Figure S4. The fitting curve of extinction coefficient of CNs at 808 nm. Figure S5. The standard curve of DOC measured by HPLC. Figure S6. In vitro NIR-I controlled phase transition of HPDC NPs. Microscopic images of HPDC NPs at different times irradiation, including A pre-irradiation, B 2 min post-irradiation, and C 5 min post-irradiation (1 W cm−2). Figure S7. Flow cytometry analysis of DiI-labeled excess PDC NPs and FITC labeled antibody after incubation for 2 h through covalent coupling reaction. Figure S8. Fluorescence intensity of DiI-labeled HPDC NPs (100 ppm) in Walker256 cancer cells after different incubation durations (n = 3 per group). Figure S9. The percentage of live, early apoptosis and late apoptosis cells in different treatment groups. (1: control (treated with PBS), 2: Laser only, 3: HPDC only, 4: DOC only, 5: HPC + 808 nm laser, and 6: HPDC + 808 nm laser groups). Figure S10. GO enrichment analysis of SDEGs. Figure S11. The pathway map of PPAR signaling pathway. Yellow marked nodes are associated with up-regulated enriched genes, blue marked nodes are associated with down-regulated enriched genes, and green nodes have no significance. Figure S12. A In vivo PA images in lymph tissues after subcutaneous injection of PDC NPs at varying time intervals. B PA signal intensity values at lymph regions after varied treatment durations (n = 3 per group). Figure S13. Skin appearance and H&E stained images after 24 h of subcutaneous injection with PBS, HPDC NPs and DOC, respectively (Scale bars: 50 μm). [file 12951_2021_1186_MOESM1_ESM.docx]

**Supporting Information**

**A novel targeted multifunctional nanoplatform for visual chemo-hyperthermia synergy therapy on metastatic lymph nodes via lymphatic delivery**

Weiwei Liu^1*^, Xiaoping Ye^1*^, Lingyun He^1*^, Juan Cheng^1^, Wenpei Luo^1,2^, Min Zheng^1^, Yaqin Hu^1^, Wei Zhang^1^, Yang Cao^1^, Haitao Ran^1†^, and Lu Yang^1,2†^

^1^Chongqing Key Laboratory of Ultrasound Molecular Imaging, Institute of Ultrasound Imaging, Department of Ultrasound, The Second Affiliated Hospital of Chongqing Medical University, Chongqing 400010, P. R. China.

^2^Department of Breast and Thyroid, The Second Affiliated Hospital of Chongqing Medical University, Chongqing 400010, P. R. China.

^*^These authors contributed equally to this work.

^†^Correspondence: ranhaitao@hospital.cqmu.edu.cn; [yanglu@hospital.cqmu.edu.cn](mailto:yanglu@hospital.cqmu.edu.cn)

Full list of author information is available at the end of the article.

**Supplementary Figures**


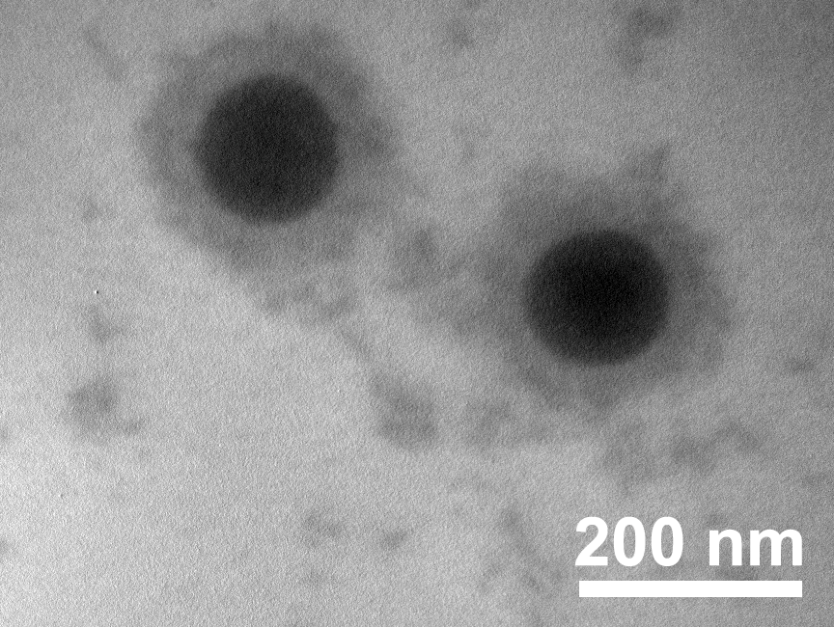


**Figure S1.** TEM image of CNs-PLGA NPs (denoted as CNP NPs).


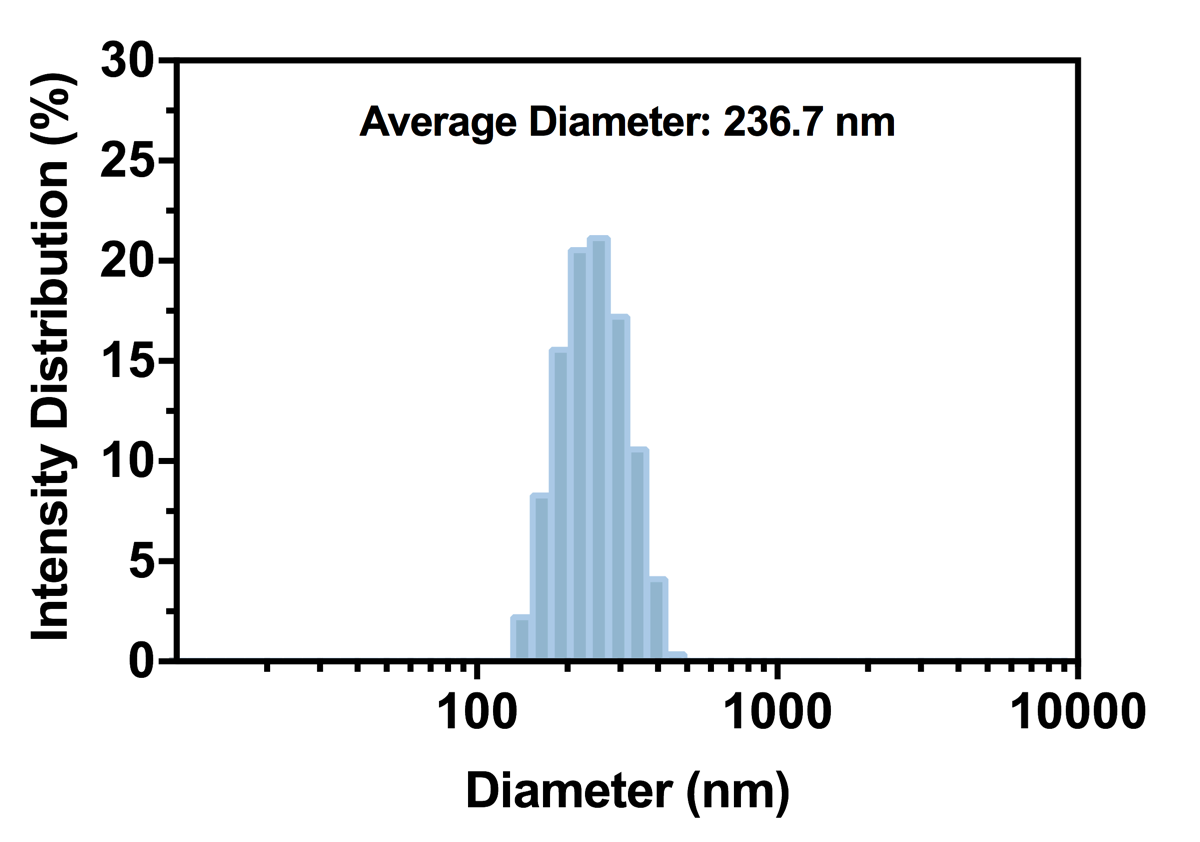


**Figure S2.** Size distribution of CNP NPs as measured by DLS.


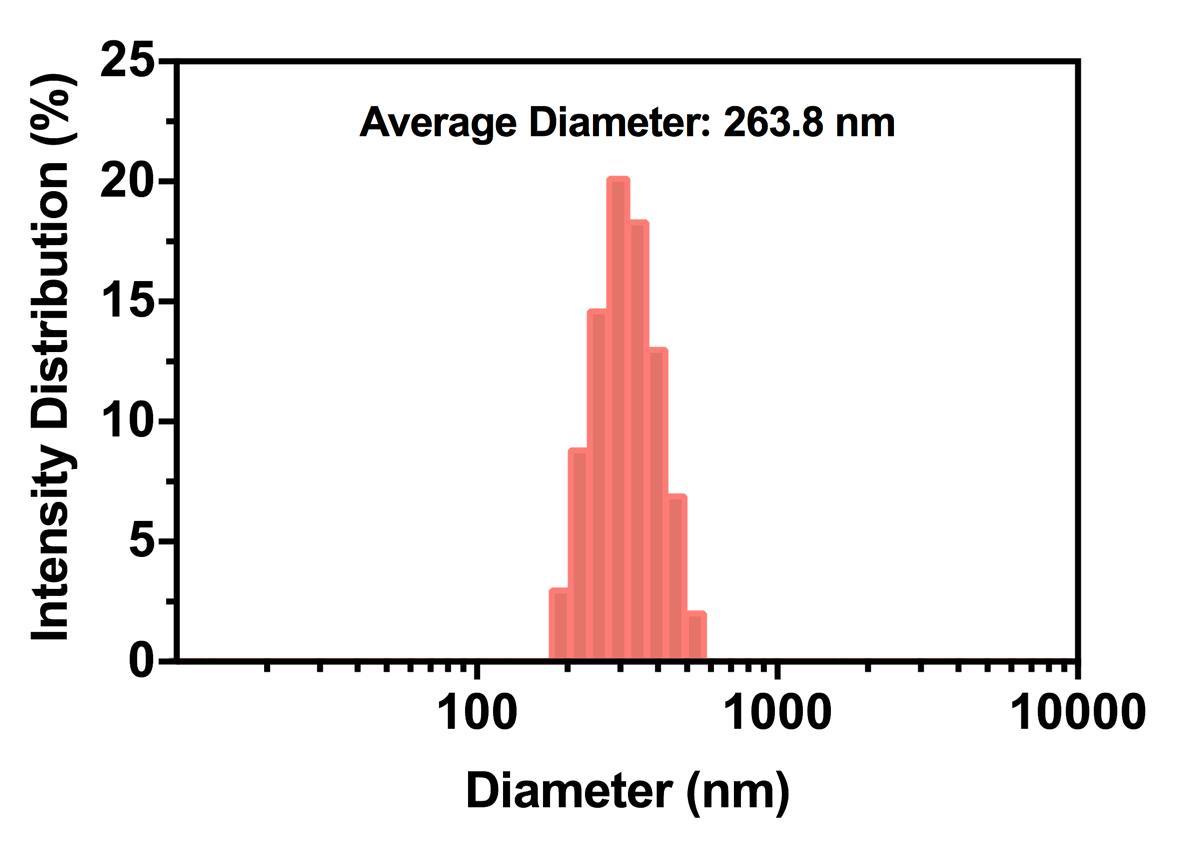


**Figure S3.** Size distribution of PDC NPs as measured by DLS.


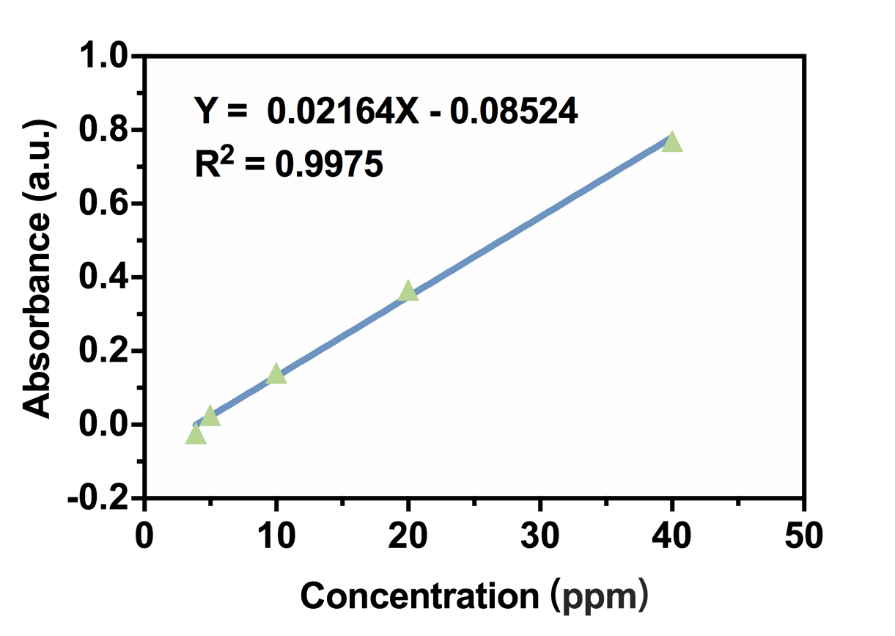


**Figure S4.** The fitting curve of extinction coefficient of CNs at 808 nm.


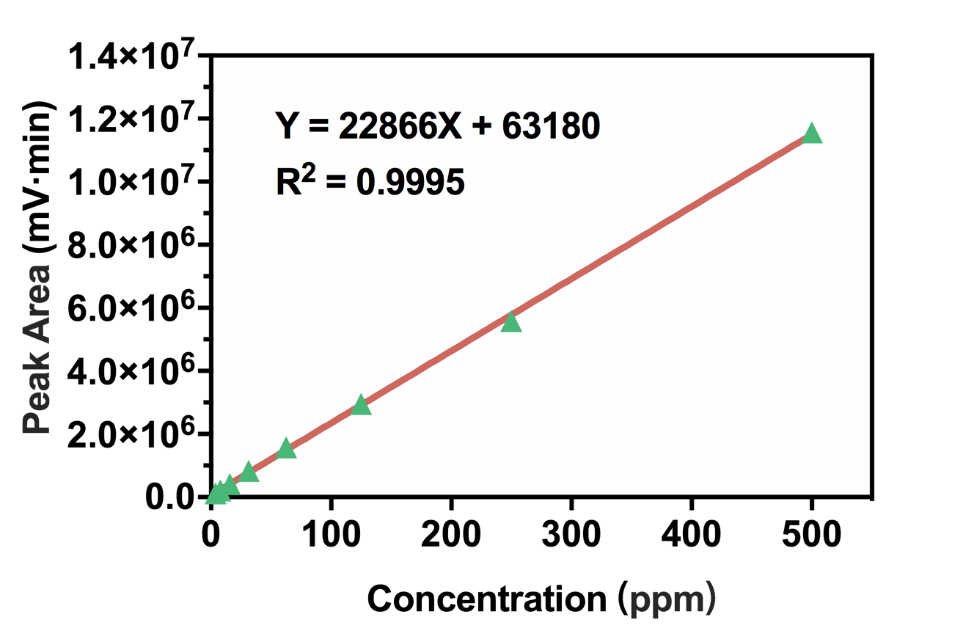


**Figure S5.** The standard curve of DOC measured by HPLC.

**
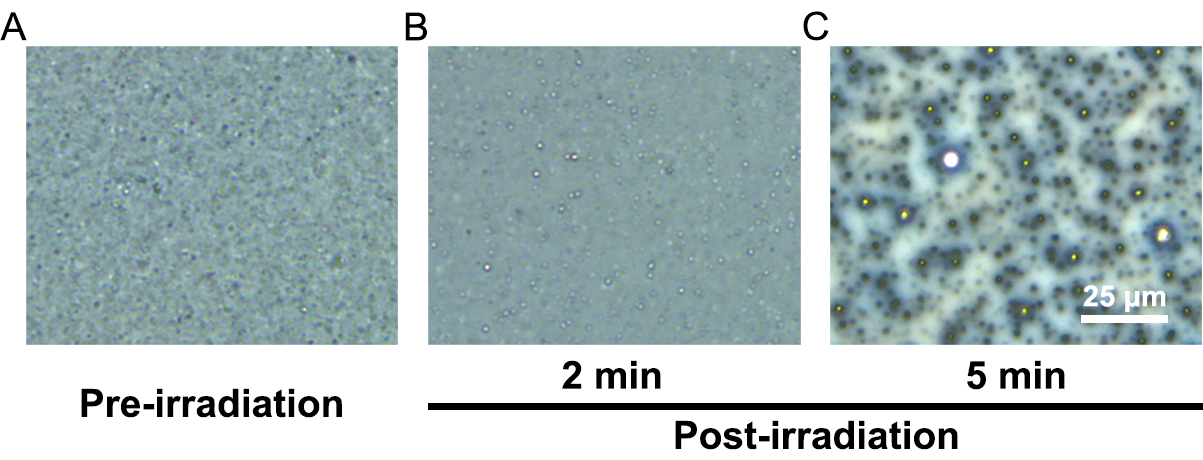
**

**Figure S6.** ***In vitro* NIR-I controlled phase transition of HPDC NPs.** Microscopic images of HPDC NPs at different times irradiation, including **A** pre-irradiation, **B** 2 min post-irradiation, and **C** 5 min post-irradiation (1 W cm^−2^).


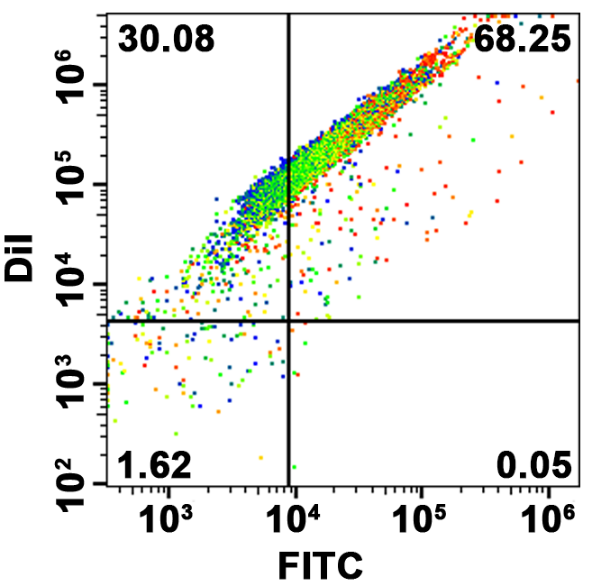


**Figure S7.** Flow cytometry analysis of DiI-labeled excess PDC NPs and FITC labeled antibody after incubation for 2 h through covalent coupling reaction.


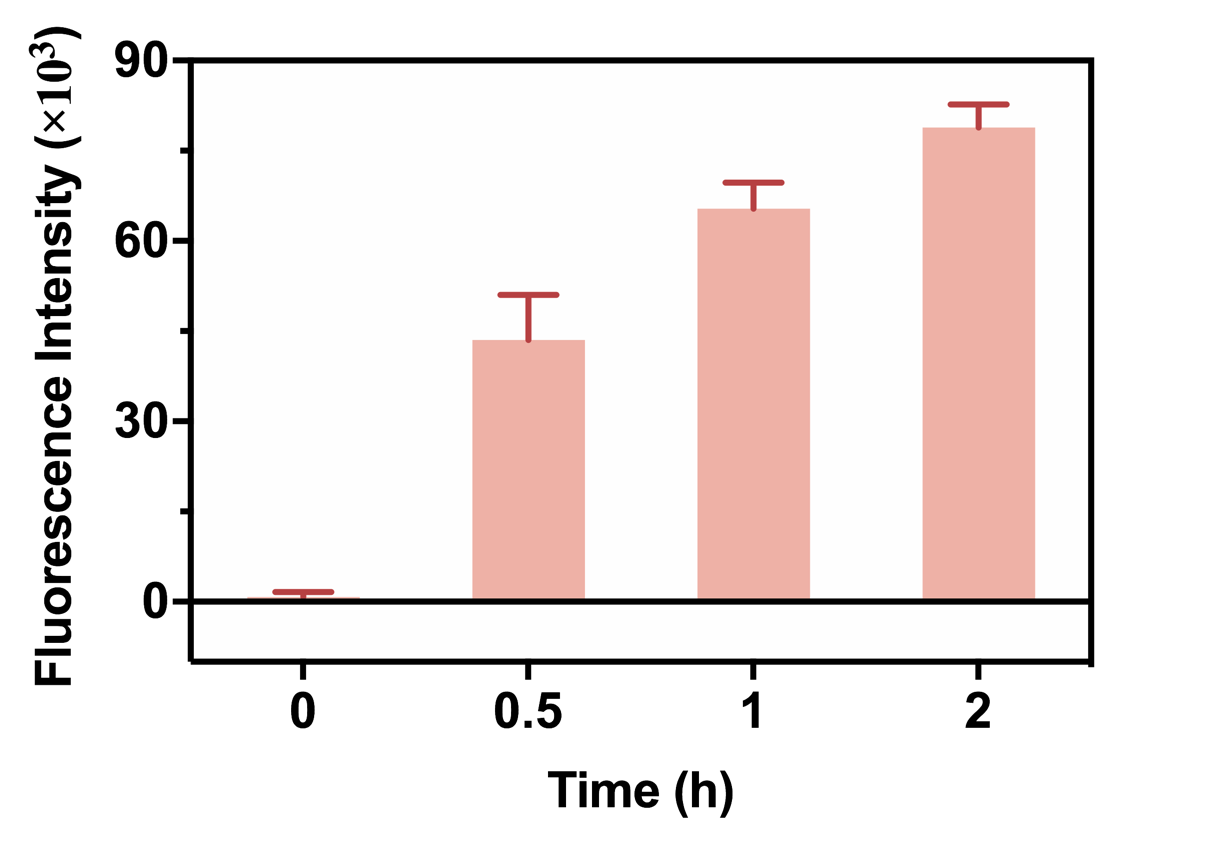


**Figure S8.** Fluorescence intensity of DiI-labeled HPDC NPs (100 ppm) in walker256 cancer cells after different incubation durations (n = 3 per group).


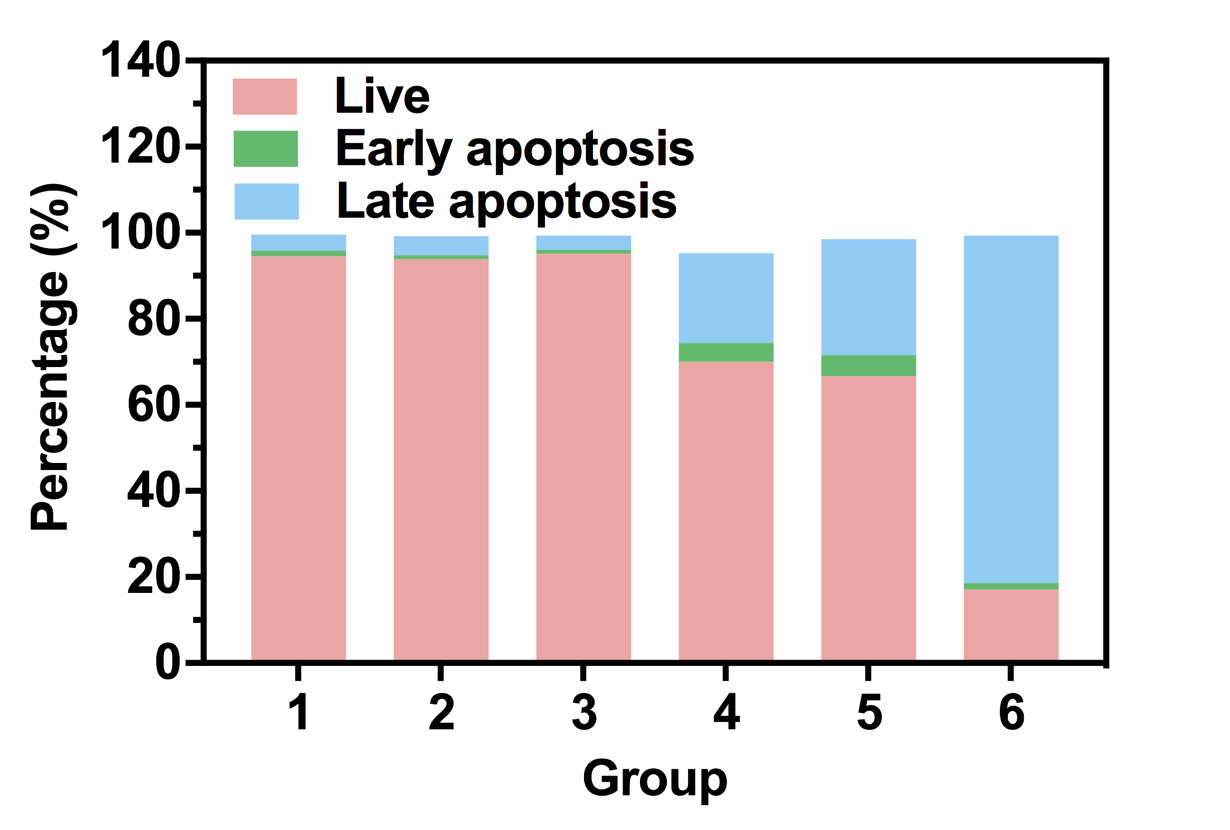


**Figure S9.** The percentage of live, early apoptosis and late apoptosis cells in different treatment groups. (1: control (treated with PBS), 2: Laser only, 3: HPDC only, 4: DOC only, 5: HPC + 808 nm laser, and 6: HPDC + 808 nm laser groups.)

**Figure S10.** GO enrichment analysis of SDEGs.


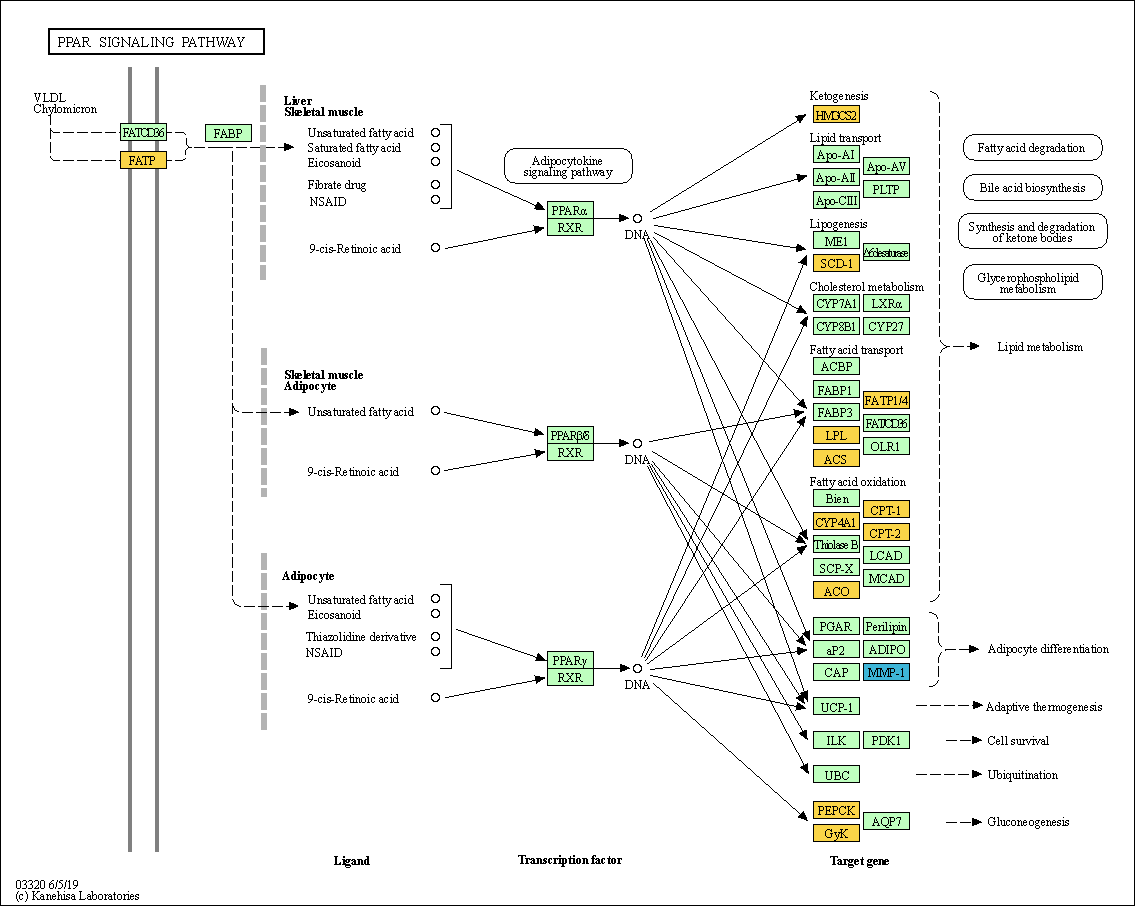


**Figure S11.** The pathway map of PPAR signaling pathway. Yellow marked nodes are associated with up-regulated enriched genes, blue marked nodes are associated with down-regulated enriched genes, and green nodes have no significance.


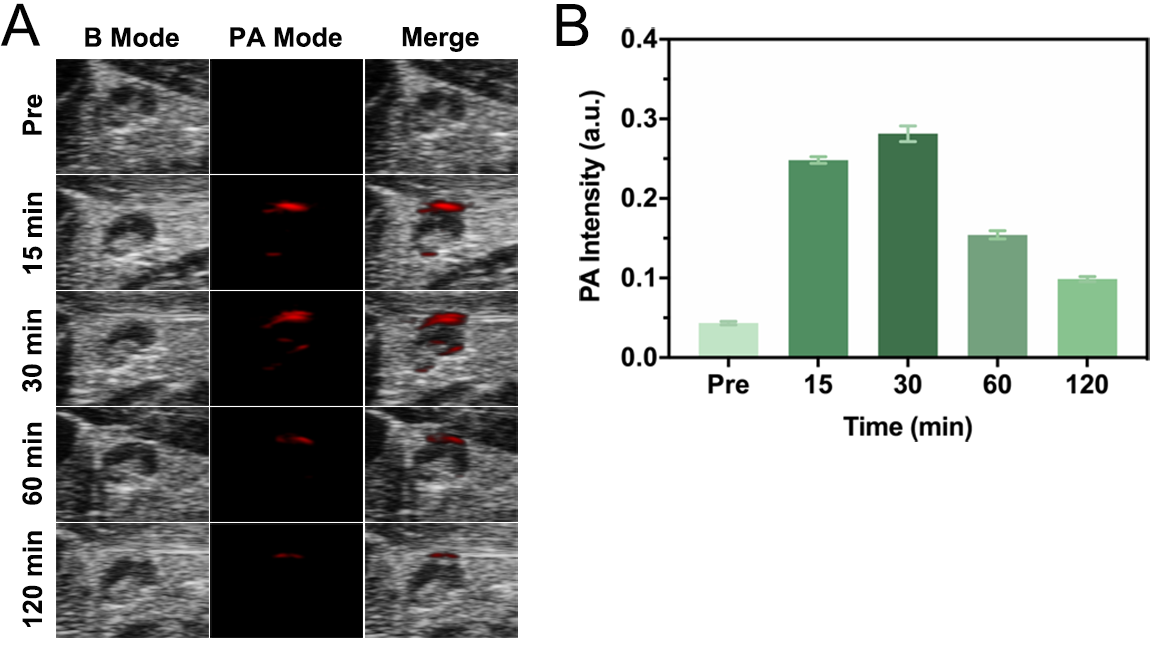


**Figure S12. A** *In vivo* PA images in lymph tissues after subcutaneous injection of PDC NPs at varying time intervals. **B** PA signal intensity values at lymph regions after varied treatment durations (n = 3 per group).


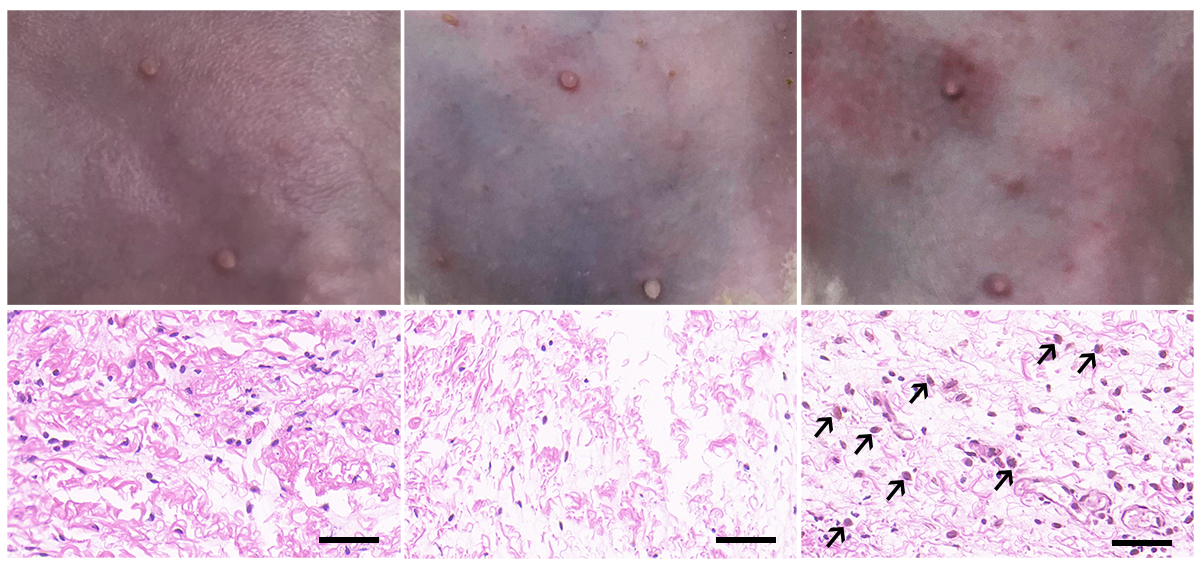


**Figure S13.** Skin appearance and H&E stained images after 24 h of subcutaneous injection with PBS, HPDC NPs and DOC, respectively (Scale bars: 50 μm).
